# Significance of an Electrochemical Sensor and Nanocomposites: Toward the Electrocatalytic Detection of Neurotransmitters and Their Importance within the Physiological System

**DOI:** 10.1021/acsnanoscienceau.2c00039

**Published:** 2022-10-28

**Authors:** Harjot Kaur, Samarjeet Singh Siwal, Reena V. Saini, Nirankar Singh, Vijay Kumar Thakur

**Affiliations:** † Department of Chemistry, M.M. Engineering College, Maharishi Markandeshwar (Deemed to be University), Mullana-Ambala, Haryana 133207, India; ‡ Department of Biotechnology, Maharishi Markandeshwar (Deemed to be University), Mullana-Ambala, Haryana 133207, India; § Biorefining and Advanced Materials Research Center, Scotland’s Rural College (SRUC), Kings Buildings, Edinburgh EH9 3JG, United Kingdom; ∥ School of Engineering, University of Petroleum & Energy Studies (UPES), Dehradun, Uttarakhand 248007, India; ⊥ Centre for Research & Development, Chandigarh University, Mohali, Punjab 140413, India

**Keywords:** Electrochemical sensors, neurotransmitters, dopamine, physiological system, voltammetry technique, electrochemical detection, metal nanoparticles, conducting polymers, impedance technique

## Abstract

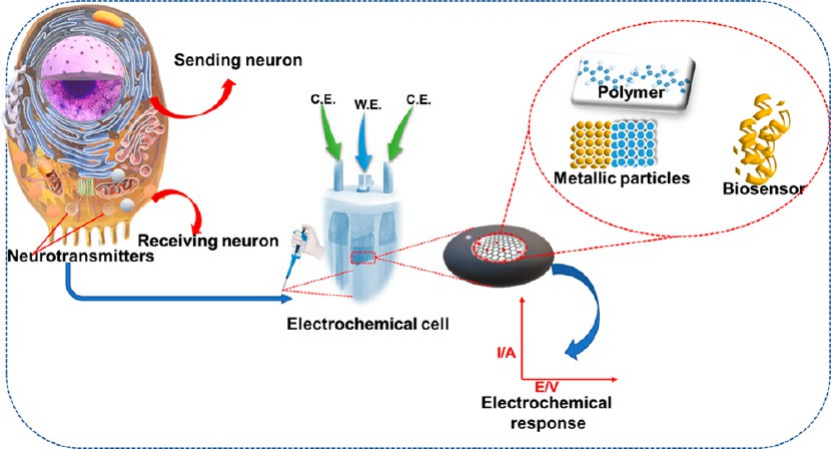

A prominent neurotransmitter (NT), dopamine (DA), is a chemical messenger that transmits signals between one neuron to the next to pass on a signal to and from the central nervous system (CNS). The imbalanced concentration of DA may cause numerous neurological sicknesses and syndromes, for example, Parkinson’s disease (PD) and schizophrenia. There are many types of NTs in the brain, including epinephrine, norepinephrine (NE), serotonin, and glutamate. Electrochemical sensors have offered a creative direction to biomedical analysis and testing. Researches are in progress to improve the performance of sensors and develop new protocols for sensor design. This review article focuses on the area of sensor growth to discover the applicability of polymers and metallic particles and composite materials as tools in electrochemical sensor surface incorporation. Electrochemical sensors have attracted the attention of researchers as they possess high sensitivity, quick reaction rate, good controllability, and instantaneous detection. Efficient complex materials provide considerable benefits for biological detection as they have exclusive chemical and physical properties. Due to distinctive electrocatalytic characteristics, metallic nanoparticles add fascinating traits to materials that depend on the material’s morphology and size. Herein, we have collected much information on NTs and their importance within the physiological system. Furthermore, the electrochemical sensors and corresponding techniques (such as voltammetric, amperometry, impedance, and chronoamperometry) and the different types of electrodes’ roles in the analysis of NTs are discussed. Furthermore, other methods for detecting NTs include optical and microdialysis methods. Finally, we show the advantages and disadvantages of different techniques and conclude remarks with future perspectives.

## 1 Introduction

To manage and control human diseases, the need for efficient and rapid diagnosis is swiftly increasing.^1^ The surging number of patients with various diseases and disorders is attributed to the continuously growing human population.^2^ Society is also subject to novel sickness stresses, disease vulnerability, and well-being syndromes associated with ecological contamination.^3−5^ Because of the extensive processes for analysis, large volumes of samples are needed to process that may lead to economic loss and deaths. Consciousness and action of the human brain come from the activities of neurons, as the human brain comprises billions of neurons. Neurotransmitters (NTs) are chemical species which serve as messengers of information between neurons.^6^ Therefore, NTs are crucial for the proper functioning of the human brain and disease diagnosis. Owing to the importance of NTs in pathological research, their sensitive and selective detection with high accuracy in real-life samples is highly desirable.^7,8^ Existing technologies can assess and diagnose patients; however, a few drawbacks also exist. Conventional analysis methods require specialized laboratory professionals to conduct analysis and are very time-consuming. With the surging advancement of science and technology, the demand for tailored medication and point-of-care (POC) strategies has increased. A scientific diagnostic tool can constitute this newly developed analytical process. Currently, researchers focus on the improvement of analytical techniques to make them more rapid, user-friendly, and miniaturized.^9^

The worldwide known glucose sensor is a POC diagnostic device that determines glucose levels within human beings and has established a multibillion-dollar market across the globe. It is an electrochemical-based sensor. Consequently, we can say that electrochemical-based sensors show immense potential due to the optimizable and modified properties of diagnoses.^10,11^ These sensors are quantitatively or semiquantitatively able to convert the information concerning the occurrence of a composite or particle in a sample within valuable analytical signals. The receptor and transducer are two critical components of electrochemical sensors. Receptors comprise active sensing materials such as nanoparticle-based composites. Conductive polymers exist within the receptor. The former has outstanding intrinsic properties such as high selectivity, sensitivity, and specificity to detect NTs. At the same time, the latter possesses excellent redox properties. In conjunction, they can detect electrochemically active molecules successfully. The chemical events within the system can be converted into analytically sound signals by the transducer.^12,13^

During the past few years, for the detection of NTs, numerous electrochemical sensors have been designed.^14−16^ The top priority related to electrochemical sensors is the modification of the working electrodes (WE) due to the vulnerability of fouling that affects the electrochemical tendencies of the entire WE. With modification, the electrocatalytic performance of various WEs like glassy carbon electrodes (GCEs), ubiquitous gold, screen-printed, platinum, and indium tin oxide (ITO) electrodes can be improved to fabricate electrocatalytic sensors having distinct sensitivity and selectivity. When we incorporate nanomaterials like carbon nanotubes (CNTs) and carbon nanofibers (CNFs) onto bare electrodes, such as Ag, Pt, Au, and GCE, it improves the ability of electrochemical detectors in terms of sensitivity and selectivity.^17−19^

Carbon-based nanomaterials can be considered as outstanding materials for the incorporation in the area of biosensing to detect NTs,^20^ agricultural pollutants,^21^ and pharmaceutical pollutants.^22^ Here, in this review article, we summarized all of the insight views on history, the significance of an electrochemical sensor and nanocomposites toward the electrocatalytic detection of NTs, and possible aspects. Per our knowledge, this is the first review where readers can go through a brief overview of the study and explanation of NTs and important analytes/NTs within the physiological system (such as dopamine, epinephrine, serotonin, norepinephrine (NE), l-glutamate, γ-aminobutyric acid, and acetylcholine). Furthermore, we discuss the history and overview of electrochemical sensors with the role of incorporation of the electrode surface (such as polymers, metallic particles, composite materials, and carbon-based materials). Additionally, we discuss electrochemical sensors and the corresponding techniques (voltammetric, amperometry, impedance, and chronoamperometry) and the different roles of electrodes in the analysis of NTs. Furthermore, other methods for detecting NTs include optical and microdialysis methods. Finally, we show the advantages and disadvantages of different techniques and conclude with remarks about future perspectives. Herein, we have collected much information on NTs and analyzed their importance within the physiological system.

## 2 Brief Overview on the Study and Explanation of NTs

Neurotransmission occurs through units, endogenous chemicals in the physiological system. In the operating and functioning of the CNS, these neurotransmitting species are crucial and involved in various metabolic and physiological activities. Their concentration should be monitored and regulated as these NTs exhibit repressive and excitatory effects upon numerous body functions. Any fluctuations in the concentration of NTs directly affect mood, digestion, emotions, and pain. In severe cases, their imbalance can cause various neurological diseases and disorders.

NTs are incorporated into vesicles and are bonded through the plasma membrane in a Ca^2+^ influx arbitrated procedure after stimulation. In turn, Ca^2+^ fuses to NT-filled synaptic vesicles with the presynaptic membrane^23^ and finally attaches to the receptor situated at the dendrite. It can support a force of reactions that causes particular activities within the human body. Any fluctuations in the concentration of these neurological messengers can cause numerous diseases and disorders. For instance, an abnormal DA level may cause severe ailments, such as Parkinson’s disease (PD), schizophrenia, and Alzheimer’s disease.^24^ Therefore, detecting and regularly analyzing these fluids within biological systems is crucial. Electrocatalytic sensors are excellent because they possess good sensitivity, controllability, quick reaction, and real-time recognition.^25^ The electroactive behavior of the electrochemical compartment allows the oxidation of the NTs and a current rejoinder associated with the recognition of the NTs.

For the proper functioning of numerous physiological systems, micronutrients are also essential. Iodine is a micronutrient^26^ that is an indispensable biological element. It is related to various functions such as cell growth, thyroid-related systems, and neurological systems.^27,28^ In the thyroid gland, iodine plays a crucial role in constructing thyroid hormones like triiodothyronine (T3) and thyroxine (T4). Many metabolic functions can be controlled using these hormones.^29^

**Figure 1:**
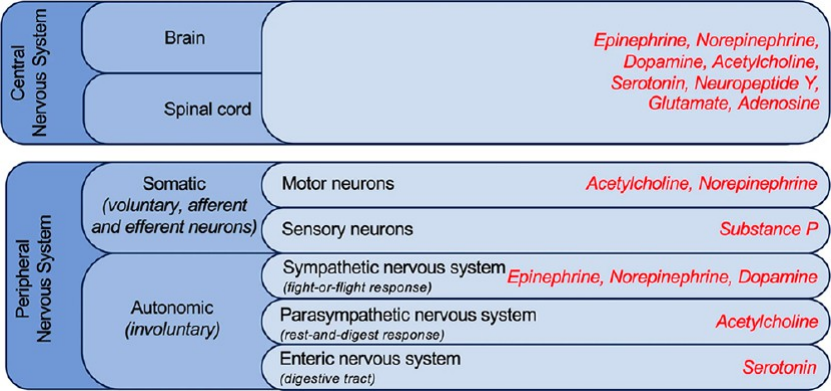
Schematic diagram showcasing top parts of the human nervous system, and the red color represents neurotransmitters released by each part. Reprinted with permission under a Creative Commons Attribution 4.0 International License from ref (30). Copyright 2020 Nature.

The nervous system structure is separated into central and peripheral nervous systems (PNS), accountable for coordination, movements, feelings, and processing. The brain and spinal cord are part of the CNS that coordinate the entire body’s activities, which means it coordinates the body’s movement; emotions, thoughts, and sensations can be experienced through these parts. All neurons that occur exterior of the brain and spinal cord are parts of the PNS and connect the CNS to whole body parts. Long nerve fibers, as well as ganglia, are included in the PNS. This system is separated into two parts: the autonomous nervous system (ANS) and the somatic nervous system (SMS). The former is responsible for the involuntary function. At the same time, the latter controls voluntary movements (Figure 1).^30^

With an imbalance of these analytes, various diseases and disorders may develop. World Health Organization (WHO) revealed that nearly 1 billion and 6.8 million people were affected and died, respectively, in 2007, due to neurologically related disorders and diseases.^31^ The imbalance metabolism of iodine may cause severe health issues such as goiters, hypothyroidism, and hyperthyroidism.^26,28^

Therefore, there is a requirement to develop and improve detection techniques. High sensitivity, selectivity, and cost-effective detection methods should also be user-friendly. For physiological and neurochemical systems, electroanalytical chemistry is proven to be an alternating analytical branch. Electrochemical-based sensors are a lucrative research avenue in analytical terms as they possess great sensitivity, rapid responses, easy controllability, and real-time detection.^32,33^ Owing to the complexity of biological systems, developing such devices remains challenging.

Conductive polymers (CP) as conjugated systems are regularly utilized in various applications such as light release, biochemical sensors, and electronic tools owing to their better catalytic performance, successful synthesis, and good ecological steadiness. Due to cost-effectiveness, electrical and chemical steadiness, and the scope of incorporating the functional groups, polyaniline (PANI) and its derivatives are very helpful in biosensory development.^34^ By doping these CPs with metal nanoparticles, the conductivity will substantially increase. In an in situ process, composite formation and polymerization occur concurrently to counterbalance the positive charge forced on polymer chains, in which a metal nanoparticle inserted onto it acts as an anion.^35^ This polymer matrix prevents coagulation by supporting and stabilizing the metal nanoparticles.^36^

Because of size-dependent optical and electrical characteristics, noble metal particles like Ag, Au, and Pt significantly impact biosensing. They possess admirable characteristics for biomolecular detection, with improved electronic indication transduction. Nanotechnology helps improve the substance’s detection capability by enhancing the noble materials’ surface area. With the help of accelerated electron transmission, it allows fast diffusion to target molecules that enhance current response.^37,38^

The capability to physiologically govern the absorption of NTs could benefit the strategy of therapeutics and the assessment of therapeutic efficacy to comparative illnesses and syndromes.^39^ Therefore, to attain adequate sensing, the growth and optimization of new modified electrodes will continue as paramount goals.

## 3 Important Analytes within the Physiological System

### 3.1 Iodine Deficiency and Its Significance

**Figure 2:**
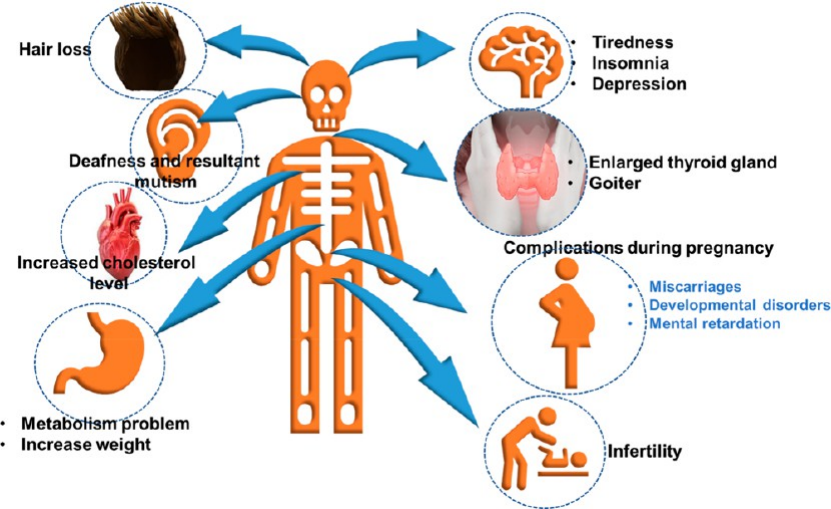
Symptoms of iodine deficiency in the human body.

Iodine is an integral organic component important in various biological movements within the human body. These functions are associated with the neurological structure, cell development, metabolism into the humanoid body, thyroid connection, and mind processes.^40,41^ Iodide being a micronutrient is implicated in the function of thyroid hormones, for example, T3 and T4 in the thyroid secretory organ. Iodothyronine deiodinase and the Na/iodide indications are thyroid proteins liable for the proficient consumption of nutritional iodide within animals.^42,43^ Essential iodine can be used in various applications in analytical chemistry, including the amalgamation of a few organic compounds and in industrial colorants and medication. Figure 2 shows iodine deficiency and its related indications when patients suffer from a life-threatening iodine inequity.

It is recommended that regular iodine consumption should be 150 μg/day. Most of the human body’s total iodine (greater than 90%) is in urine. For epidemiological studies of iodine supplementation, it is essential to determine the concentration of urinary iodine, which helps in diagnosis and regulatory iodine shortages. Urinary iodine concentrations are valuable in diagnosing temporary thyroid dysfunction and iodine-caused hyperhidrosis. According to WHO, a person with average urinary iodine absorption of around 100 μg/L (almost 0.8 × 10^–6^ M) is considered to be iodine-deficient, while patients who suffer from iodine-excess hyperthyroid will have urinary iodide absorptions that are 10- to 100-times higher than those in healthy people.^44,45^

There have been various strategies to detect and determine iodide concentration in an aqueous media. The most adept one was the Sandell-Kolthoff reaction, a spectrophotometric detection method. During this reaction, As^3+^ reduced the yellow Ce^4+^ to colorless Ce^3+^ in the presence of iodide. However, other techniques that possess good selectivity and sensitivity have been used and developed to detect iodide.^46,47^ These approaches comprise electrochemical recognition, gas chromatography, capillary electrophoresis, and optical spectrometry.

However, these methods require particular operating skills, are time-consuming, and have tedious sample preparation. However, some of these approaches are extremely sensitive and need multistep and complicated sample preparation. Only a few reports exist to determine iodine in complex biological samples due to troubles related to matrix interferences. Also, performances of a few techniques are hindered by coexisting anions. In recent times, chromogenic and fluorogenic chemosensors have been developed for sensing iodide and cyanide ions.^48^ However, the challenge remains to find a suitable anion-selective sensor.^49^ Several optical-based methods have been established to distinguish iodine ions using nanoparticles (NPs) and small molecules.

### 3.2 NTs within the Physiological System

NTs are signaling substances that influence several aspects of neuronal activities by not being transported into the blood and stored and released from a neuron. These transmitters control the physiological actions of humans by regulating communication within the neural network. Furthermore, NTs enable the proper functioning of the human nervous system, like nervous system homeostasis, behavior, and body movement. In neurotransmission, first through presynaptic neuron synthesis of a NT, then the binding and initiation of the receptor occur after the growth and discharge of the NT within the synaptic branch. Finally, the reuptake from the synapse and its mitigation occurs.^50^

NTs affect numerous neurophysiological functions such as learning, sleeping, appetite, and memory. For instance, DA is one of the crucial NTs within the CNS as it controls movement and combines characteristics such as behavior, cognitive functions, and attention-related processes.^50,51^ Any fluctuations in the concentration of NTs can cause neurodegenerative illnesses, for example, drug dependence or depression disorders.^52^ To identify and estimate pharmacodynamics and therapeutic impacts of psychiatric and neurological disorders, identifying the absorption of the plasma catecholamine and its metabolites is regularly used. Consequently, researchers are interested in the growth of effective electrochemical sensors with modified electrodes as they can control the concentration of NTs into actual models. Additionally, employing an in situ system and removing the pretreatment step will overcome the limitations of conventional methods like liquid chromatography,^53^ chemiluminescence,^54^ and electrophoresis^55^ which are laborious and time-consuming and require actions toward sample synthesis.^56^

#### 3.2.1 DA’s Importance within the Physiological System

**Figure 3:**
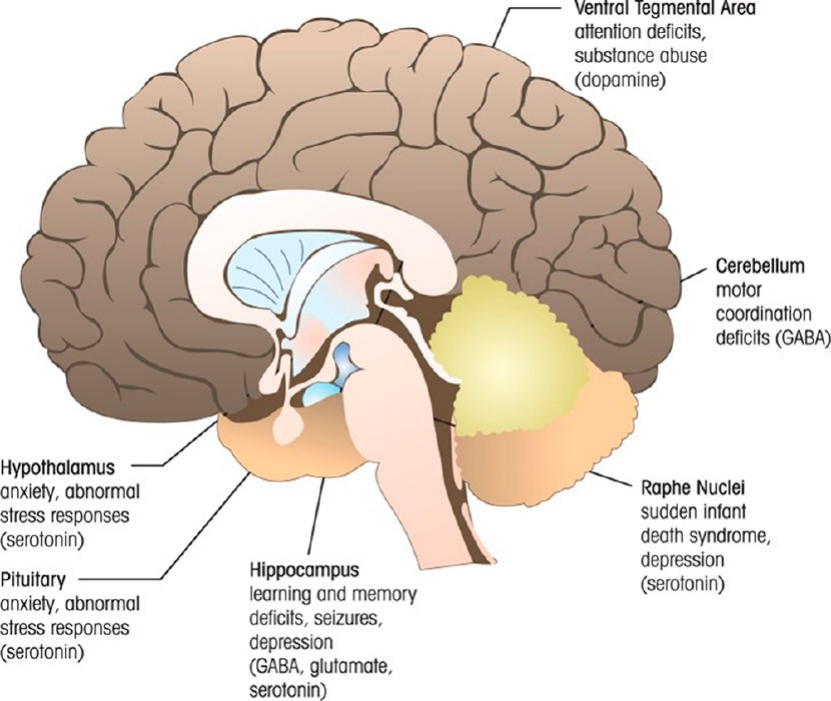
Systematic demonstration of the matured human brain and its regions where different neurotransmitters affect its functioning. Reprinted with permission under a PMC PubMed Central from ref (57). Copyright 2011.

DA is a catecholamine having a crucial role in the hormonal, renal, cardiovascular systems and the CNS. DA neurons strongly influence brain functions, including emotions, attention, movement, reward, and motivation within the mature brain, as shown in Figure 3.^57^ DA also regulates multiple functions outside of the brain. DA increases urine output in the kidneys and regulates blood sugar by reducing insulin production in the pancreas. It acts as a vasodilator, which allows blood to flow more easily. It sends inhibitory signals to the stratum.^58^ The disturbance in DA metabolism can result in numerous diseases such as epilepsy, disorientated dementia, and HIV infection.^59,60^ It mainly causes severe neuropsychiatric-related disorders like attention deficit disorder, PD, and schizophrenia, where the latter two are directly connected to the presence and absence of DA.^61^ It also plays numerous essential roles in brain regulation, neuronal flexibility, and stress control effects.^62,63^ Therefore, the detection of this analyte is very crucial.

##### 3.2.1.1 Parkinson’s Disease

PD represents a nervous system syndrome of the CNS that affects nearly 6 and 1% of the populace over 65 and 60 years old. The fundamental medical characteristics of PD include postural instability, firmness, and resting tremors. These structures tend to rise by losing around 70% of nigrostriatal dopaminergic nerve cells. Few irregular immature and young-onset PD patients have also appeared.^63^ Worldwide, 10–50/100,000 persons/year get PD, and it is estimated that this number will double by 2030.^64^

**Figure 4:**
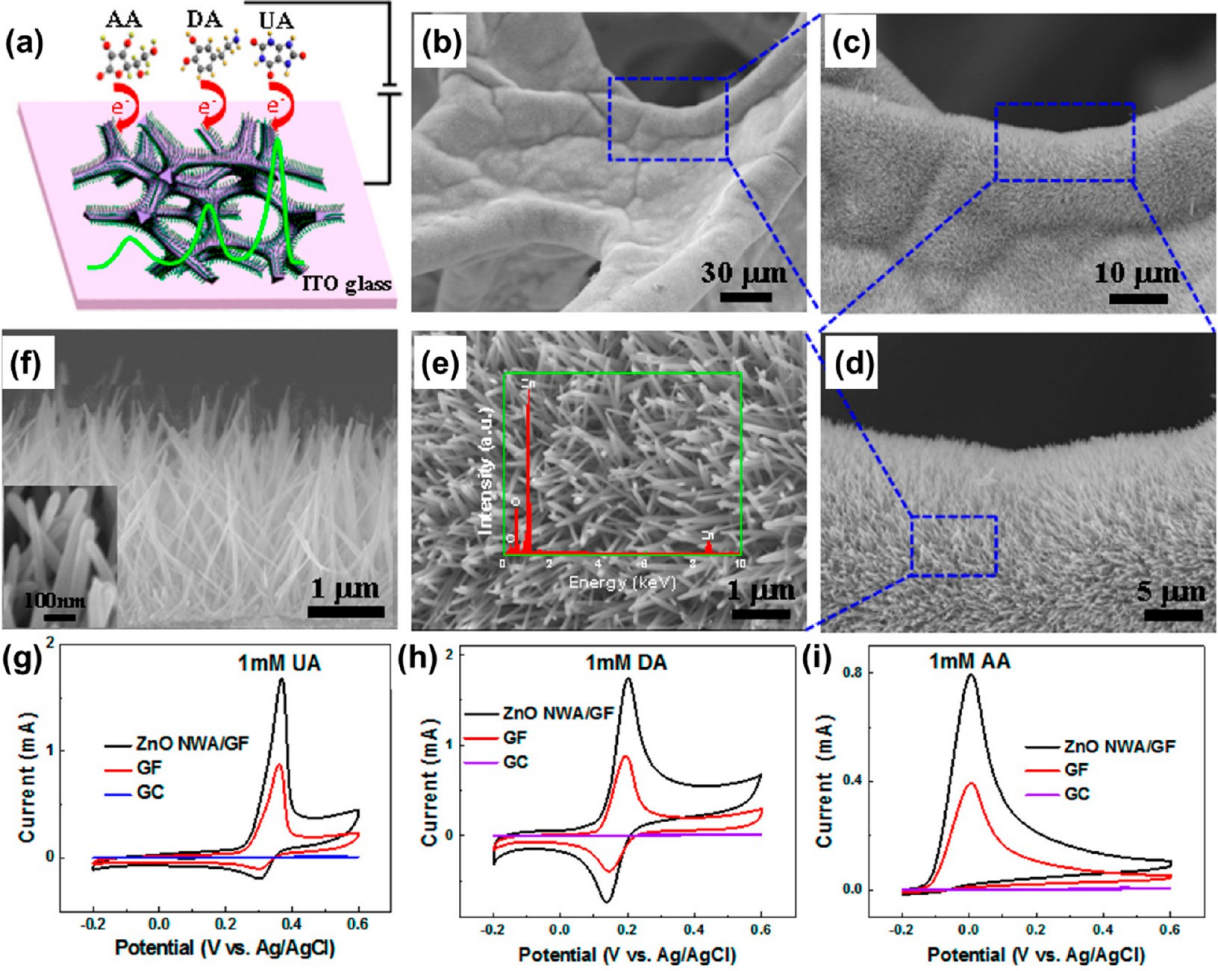
Physical investigation of the combined ZnO NWA/GF. (a) Graphic representation of the ZnO NWA/GF probe and UA, DA, and AA detection. (b–e) SEM pictures of the ZnO NWAs at the 3D GF on special intensifications. Inset: Energy-dispersive X-ray spectroscopy (EDX) of the ZnO NWAs. (f) SEM pictures of the size of the ZnO NWAs, ∼2 μm. Inset: Diameter of the ZnO NWAs, ∼40 nm. Electrochemical recognition of UA, DA, and AA. (g–i) CV arcs of the ZnO NWA/GF, GF, and bare GCE within 1 mM UA, DA, and AA, correspondingly, at a sweep speed of 50 mV s^–1^. Reprinted from ref (65). Copyright 2014 American Chemical Society.

Herein, Yue et al.^65^ reported the hydrothermal preparation of ZnO nanowire arrays (ZnO NWAs) produced at graphene foam (GF) utilizing chemical vapor deposition (CVD). A graphic representation of the incorporated ZnO NWA/GF electrode used to identify uric acid (UA), DA correspondingly, and ascorbic acid (AA) is illustrated in Figure 4a. Figure 4b–f displays scanning electron microscopy (SEM) pictures of the ZnO NWA/GF network. GF on the base of the ZnO NWAs was muscular. The GF exterior was surrounded by steeply allied, favorably homogeneous ZnO NWAs. The ZnO NWAs were ∼40 nm wide and 2 μm in size (Figure 4f). Figure 4g–i exhibits cyclic voltammetry (CV) arcs toward different electrodes at a sweep speed of 50 mV s^–1^. The ZnO NWA/GF probe had the most elevated oxidation potential, with a reasonably narrow peak window for the recognition of 1 mM UA (Figure 4g). This tendency is comparable to that followed for DA and AA at identical concentrations but at various oxidation voltages (Figure 4h,i).

The patients suffering from PD face gradual memory loss and disturbance in behavioral activities.^66^ Due to genetic factors and modern lifestyles, people are more susceptible to neurodegenerative situations such as PD, with genes acting as a predisposing factor. However, the development of diseases cannot be determined by it.^63^ In ancient Ayurveda literature, a PD-like disease had been initially illustrated, and people suffered from this disease from the 20th century; it is not a new disease.^58^

PD originates in the midbrain area (substantia nigra (SN)) by losing half of the neurons. Due to neuromelanin, these neurons have characteristically dark pigmentation, so, within the SN, there is a lack of dark pigmentation traits among PD patients.^67^ Under normal physiological conditions, these neurons are usually responsible for producing DA and forming the dopaminergic nigrostriatal area. People with dead nerve cells in the SN suffer from akinesia.^68^ Different studies on “PDs” were a complete recording of chronology, e.g., exposure to pesticides, encephalitis, certain antipsychotic medications, or the existence of specific exclusion standards or brain imaging investigations that can assist in clinching the diagnosis.^69^ While the verification of PD may be designated only through post-mortem histopathology, monitoring PD, especially early, could benefit the patients.

##### 3.2.1.2 Schizophrenia

Schizophrenia is a very prevalent psychiatric disorder that affects about 1% of humans worldwide. It is considered one of the utmost severe psychological illnesses. In Europe, it is the third most effected brain disorder after dementia.^70^ The initial symptoms are visual and auditory hallucinations and thought disorders. In contrast, in severe cases, symptoms comprise memory loss, suppressed motivation, and scarcity of executive functions.^71^ Its treatment is costly as constant care is required even if the case is reactive for the diagnosis. Its symptoms may present throughout the lifetime within patients, so it puts financial pressure on the family.

The “original DA hypothesis” states that hyperactive DA transmission results in schizophrenic symptoms. The “revised DA hypothesis” proposes hyperactive DA transmission in the mesolimbic areas and hypoactive DA transmission in the prefrontal cortex in schizophrenia patients.^72^

##### 3.2.1.3 Storing and Transportation of DA

Dopaminergic NTs are kept in synaptic vesicles in the presence of other NTs. DA particles are transported to the compartment edge when these vesicles receive an electrical stimulus that originates from nerve impulses and releases its content within the minute gap among neurite ends and the dendrite of subsequent nerve cells in the synapse. Schizophrenia and PD may stem from lower levels of DA concentration within the human body. Detecting the concentration of DA in single synapse development in biosensors is needed.^73,74^

The material is considered to be an active material to detect DA if it gives a response at a concentration lower than 1.6 mM (i.e., [DA] ≪ 1.6 mM) because the expected level of DA in the synapse is 1.6 mM. DA can be detected using electrochemical sensors because it is an electrochemically active compound.^75^

### 3.3 Epinephrine Importance within the Physiological System

A German scientist, Friedrich Stolz, shaped the primary artificial hormone in 1904, a ketone arrangement of epinephrine (known as adrenaline). In 1906, synthetic epinephrine could be synthesized on a large scale, while Stolz transformed adrenaline into adrenaline/epinephrine. The effectiveness of this synthetic hormone was announced to be favorable compared to crude adrenal extracts that had modest consequences upon sickness. In 1905, an American physiologist, Carl Wiggers, showed vasoconstrictor characteristics of artificial epinephrine on cerebral blood flow. It seemed all set for utilization as a respite drug for asthma.^76^

Epinephrine, also recognized as adrenaline, is a hormone mainly concealed by the medulla of the adrenal glands. It especially raises cardiac output and surges glucose levels in the blood. It is remarkably neutral, whereas acute stress and its stimulatory effects strengthen and make a person experience “fight or flight”.^77^ To activate the autonomic nervous system, the amygdala triggers the hypothalamus when the brain identifies any kind of danger. Epinephrine starts pumping into the bloodstream by the adrenal gland after receiving signals from the autonomic nervous system. This epinephrine increase is usually referred to as a fight or flight response or an adrenaline rush. Epinephrine affects the heart, lungs, muscles, and blood vessels. Several physiological changes such as faster breathing, increased heart rate and blood flow, and increased level of sugar in the blood occur when it is released into the bloodstream.

**Figure 5:**
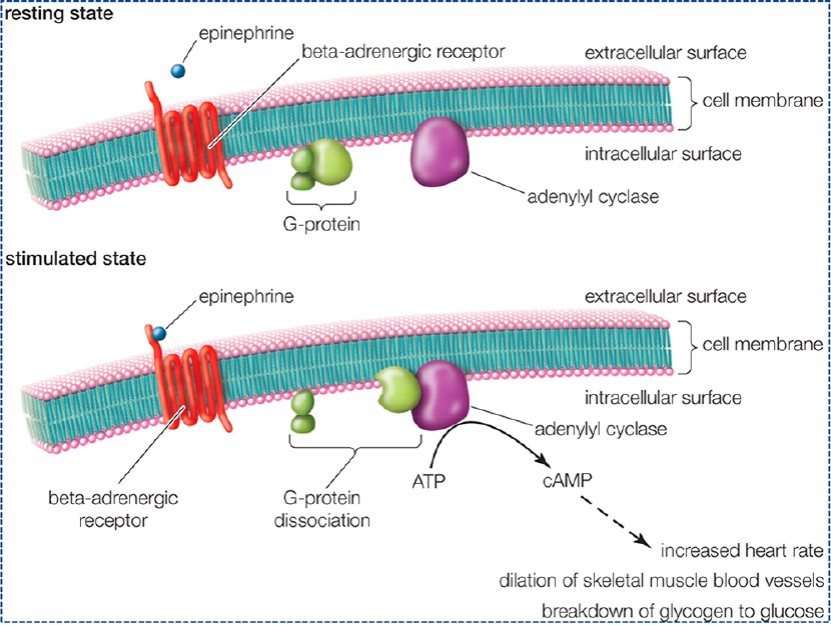
Synthesis of epinephrine-enhanced cyclic adenosine monophosphate (cAMP). The data were accessed on December 20, 2020 from ref (78) (https://www.britannica.com/science/second-messenger).

Structurally, epinephrine is almost similar to NE; the only difference is a methyl cluster on the nitrogen adjacent chain. The amine group is attached to a catechol group in the structure of both substances—a construction novel to the catecholamines. Both of these hormones are central stimulatory mechanisms of the sympathetic nervous system and are, thus, pharmacologically classified as sympathomimetic agents (Figure 5).^78^

The complex actions of epinephrine produce numerous responses due to its stimulatory properties upon α- and β-adrenergic receptors contingent at the particular receptor and tissue sites. Therefore, epinephrine causes a restriction in various minute blood vessel networks but opens the blood vessels into the lean physiques and the liver. It raises blood pressure and output by increasing heart rate and the force of heart contraction. Epinephrine excites the collapse of glycogen to glucose within the liver, resulting in growth into glucose levels within the plasma. It also increases the level of mingling free fatty acids. Our body needs more alertness and energy in stress or danger, provided by these additional sums of glucose and fatty acids. Epinephrine also contracts the dilater powers of the iris within the eye, resulting in mydriasis and enhanced visual perception.

### 3.4 Serotonin or 5-Hydroxytryptamine (5-HT) Importance within the Physiological System

Dietary proteins contain the essential amino acid l-tryptophan from which serotonin can be synthesized. Only 1% of dietary tryptophan is converted to serotonin. A two-step procedure is a sodium-dependent aromatic l-amino acid transporter transports tryptophan into the serotonergic nerve and converted tryptophan into serotonin (5-HT). The primary stage is enzymatic hydroxylation of tryptophan to 5-hydroxytryptophan (5-HTP), which takes place by tryptophan hydroxylase. The second step is the decarboxylation of 5-HTP to form 5-HT.^79^ 5-HT is intricate in several physiological events, such as sleep, thermoregulation, knowledge and memory, discomfort, (social) behavior, sex, eating, motor activity, and biological tempos.

**Figure 6:**
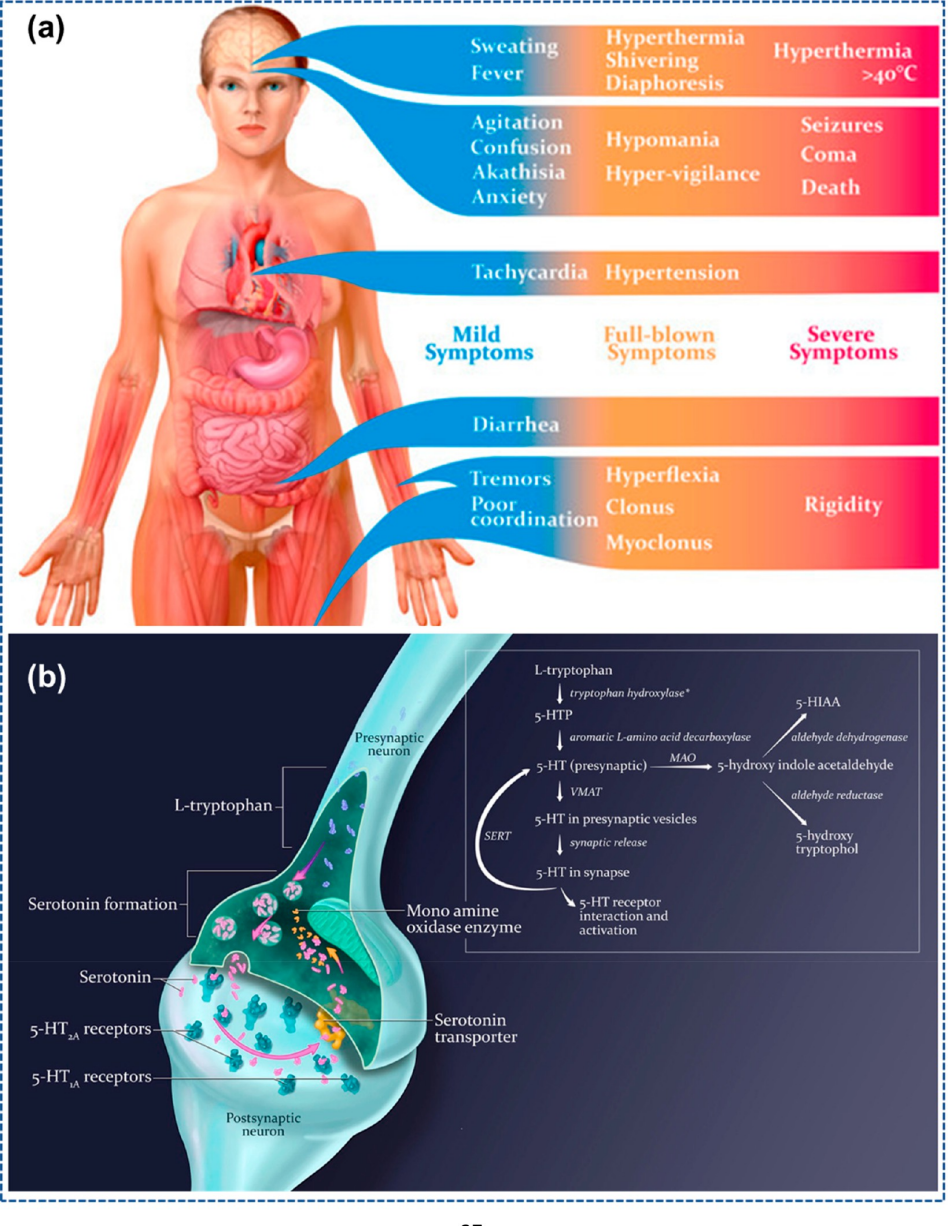
(a) Signs and symptoms that arise sideways a spectrum of severity of the serotonin syndrome. Minor symptoms can be unnoticed and considered as just more than flu-like symptoms. (b) At standard serotonergic nerve cells, 5-HT absorptions at a synapse are defined with different methods, such as preparation, regulated leakage from the presynaptic nerve cell, reuptake, and breakdown. VMAT = vesicular monoamine transporter; SERT = serotonin reuptake transporter; 5-HIAA = 5-hydroxyindole acetic acid. Reprinted with permission under an open access Creative Common CC BY license from ref (83). Copyright 2019 MDPI.

In 2003, Dunkley et al.^80^ developed the most recent criteria for its diagnosis. Dunkley’s criterion was created using a toxicology record known as the hunter zone toxicology facility that included patients overdosed with at least one serotonergic medication. A step-by-step diagram was constructed that comprises symptoms that returned statistically considerable rates in sufferers with serotonin syndrome (SS) identified with a medicinal toxicologist. This investigative scheme was more delicate (84% vs 75%) and explicit (97% vs 96%) than Steinbach’s SS diagnosing criteria. The tracker 5-HT poisonous condition, as known now, is currently considered the gold standard for typical diagnosis of this sickness.^81^ In 30% of patients, symptoms usually occur within 120 min, and in 60% of patients, it occurs within 6 h of exposure to triggering medications.^82^ In minor cases, flu-like symptoms appear, whereas it can cause cardiovascular collapse and death in critical cases (Figure 6a). The synthesis of 5-HT is showcased from the crucial amino acid l-tryptophan, obtained from nutritional protein (Figure 6b). Like phenylalanine, leucine, and methionine, other neutral amino acids present in the brain are transferred by the identical transporter as l-tryptophan.^83^

As aforementioned, the serotonergic structure is a complex system for controlling the micturition reflex; the dependence of 5-HT on the types of receptors and the target organs that are present can show opposing effects. In the clinical analysis, selective serotonin reuptake inhibitors (SSRIs) or serotonin noradrenaline RI (SNRI) that are examples of unselective serotonergic drugs may cause urinary retention or hesitancy. Under the circumstances where a patient is being prescribed these types of drugs, such as sufferers with unhappiness, care should be taken about these uncommon urinary signs.^84^

### 3.5 Norepinephrine Importance within the Physiological System

NE is a monoamine NT with a broad physiological role in the CNS and the PNS. However, there is no practical way to study the functional characteristics of a particular noradrenergic synapse in the brain. New approaches to imaging synaptic neurotransmission are essential to study the specific synaptic variations that happen during pathological processes, behavior, and learning.

**Figure 7:**
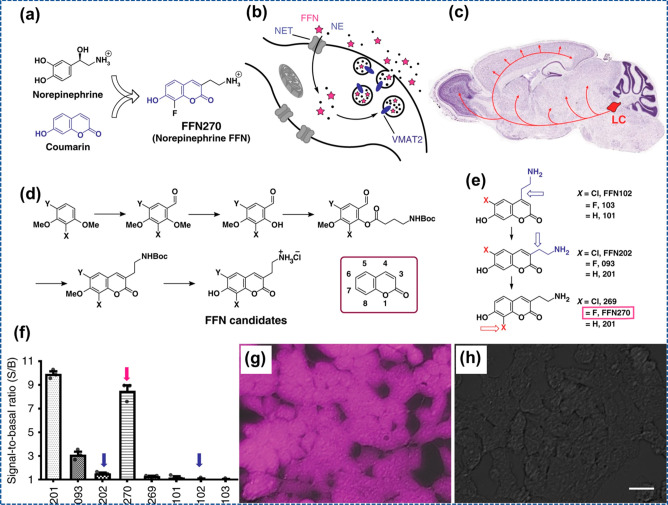
Schematic structure of NE-FFNs. (a**)** Formation of NE-FFNs with a combination of constitutional aspects of NE with the coumarin. (b) NE-FFNs delineate NE uptake from the extracellular area, wrapping within cysts, and exocytosis as they are prepared to support NET and VMAT2. (c) Figurative picture of NE neuron dispersal into the brain. (d) General artificial method toward synthesizing 3-series aminoethyl-7-hydroxy coumarins as possible NE-FFN nominees. (e) Concentration sequence of sample NE-FFNs. (f) Complete cellular fluorescence after loading FFN samples (5 μM) in human embryonic kidney cells stably transfected with human NET (hNET-HEK) cells. Example pictures of FFN270 in the absence of inhibitor (g) and the presence of inhibitor (h). Reprinted with permission under a Creative Commons Attribution 4.0 International License from ref (87). Copyright 2018 Nature.

NE, also called noradrenaline (NA), is the main neurotransmitter of the sympathetic PNS, which influences the functioning of the immune system, most visceral organs, and glands. NE is also a significant neurotransmitter of the CNS.^85^ Note that NE nerve cells initiate within locus coeruleus throughout the CNS and amygdala, cerebellum, and spinal cord.^86^ Dunn et al.^87^ introduced the false fluorescent neurotransmitter (FFN) utilization model to get the visual tracer of NE neuron communication. Numerous classes of fluorescent DA are developed in laboratories that reveal the heterogeneous nature of the presynaptic performance of DA in brain tissues of mice. FFN is one such example of FFN (Figure 7), demonstrating its selectivity as a substrate for dopaminergic neurons. It is clear that FFN102 is not a norepinephrine transporter (NET) substrate, meaning it is a poor optical tracer for NE.

Also, the FFN model’s capacity was expanded by launching probe FFN270, which is the first NE-FFN (Figure 7). It is a substrate for fluorescent NET and neuronal vesicular monoamine transporter (VMAT2). Synaptic vesicle content that is released during exocytosis is measured by this probe within NE axonal varicosities when taken up with NE. FFN270 allows an assessment of noradrenergic microanatomy and in vivo provides information regarding cortex activity of synapse of intact neuronal circuits.

During low blood pressure and stress, the adrenal medulla produces NE. NE increases blood pressure by promoting vasoconstriction (narrowing the blood vessels). NE also raises the heart rate and blood sugar levels, like epinephrine.

### 3.6 l-Glutamate Importance within the Physiological System

**Figure 8:**
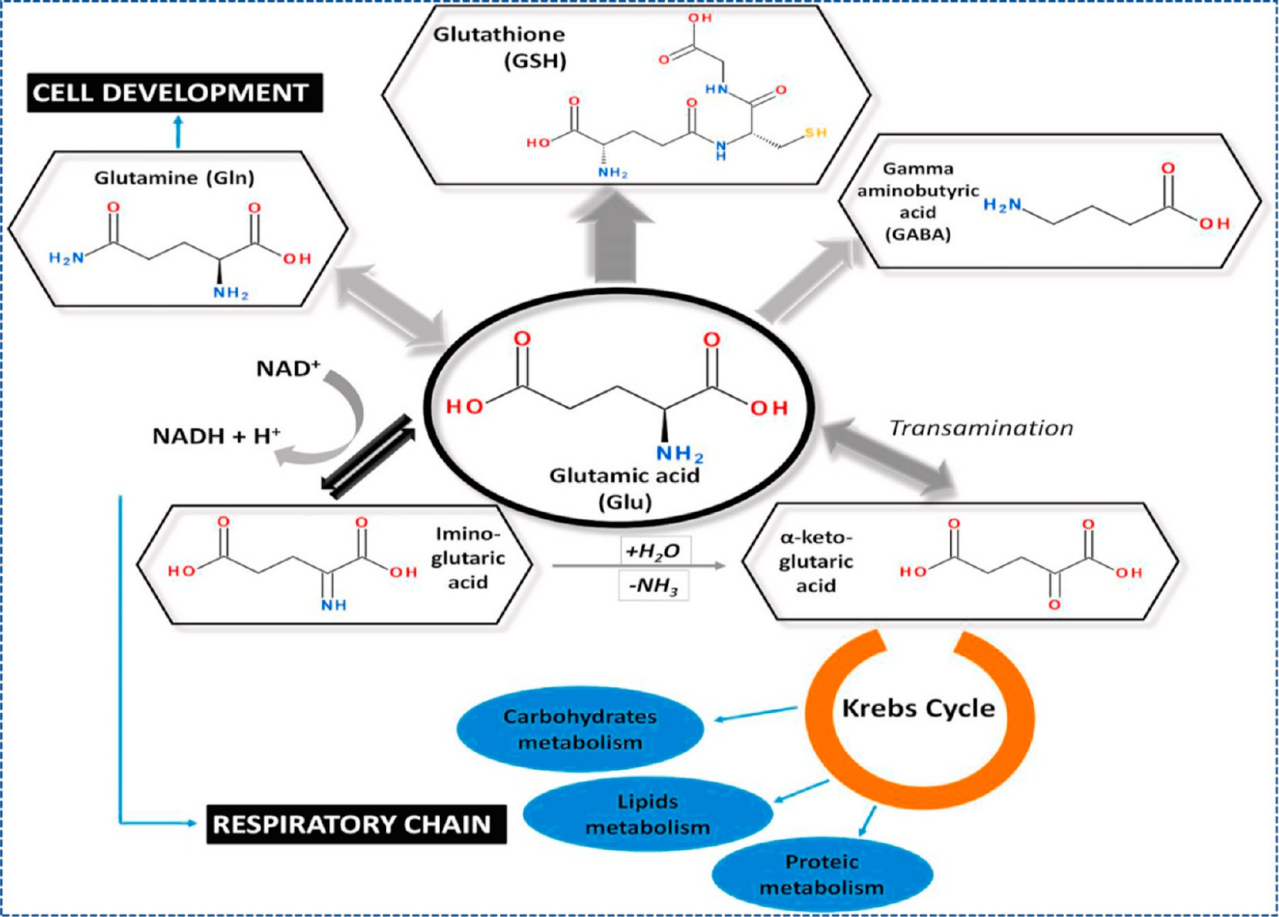
Various biological functions of Glu. Reprinted with permission from ref (88). Copyright 2021 Elsevier Ltd.

Indeed, glutamate (Glu) is an important excitatory neurotransmitter of the CNS of mammalians and has various biological functions, as described in Figure 8;^88^ however, its role in plants is not exactly known.^89^ Glu is formally categorized as a nonessential amino acid because it may be prepared (in adequate amounts for fitness) through α-ketoglutaric acid made as part of the citric acid cycle via a sequence of reactions in which the beginning point is citrate.^90^

In substantial concentrations, free Glu is an essential substrate in most organs and tissues in the renal, cardiovascular system, skeletal muscles, brain, and liver. Glu plays a vital role in producing other amino acids, glutathione, proteins, and energy metabolism. It is a primary excitatory NT in the brain. It regulates numerous activities such as learning, memory, neural development, and synaptic plasticity.^91,92^ Glu is the most productive excitatory NT within the human brain and has crucial roles in considerable brain functions and synaptic plasticity, for example, long-term potentiation. Nevertheless, extreme Glu release may be poisonous to the brain, and it may lead cells to their demise into a method now called “excitotoxicity”. Glu-mediated toxicity has been connected to multiple neurodegenerative disorders, like Alzheimer’s disease, amyotrophic lateral sclerosis, and Huntington’s disease. Glu cannot cross the blood–brain barrier independently. However, it is vigorously transported out of the nervous system by Glu transporters, maintaining its concentration in brain liquids at a reasonably stable level.^93^ Abnormalities of glutamatergic neurotransmission play an essential function in developing numerous significant psychiatric diseases.

### 3.7 γ-Aminobutyric Acid Importance within the Physiological System

γ-Aminobutyric acid (GABA) is the primary inhibitory NT within the human cortex. GABA supports the inhibitory tone that counterbalances neuronal excitation. When this equilibrium is unbalanced, outbreaks may occur.^94^ When GABA binds to its receptor, it delivers a soothing impact. This may assist with stress and nervousness and control outbreaks. Due to these characteristics, GABA has also evolved into a widespread accessory in recent years. It is partly because it is not accessible from multiple food resources. The only foods that include GABA are fermented, like kimchi, miso, and tempeh.^95^ It has been traditionally assumed that exogenous GABA (i.e., accepted as a compliment) does not transit the blood–brain border; nevertheless, data received from more recent investigations suggest that it can be feasible.^96^

**Figure 9:**
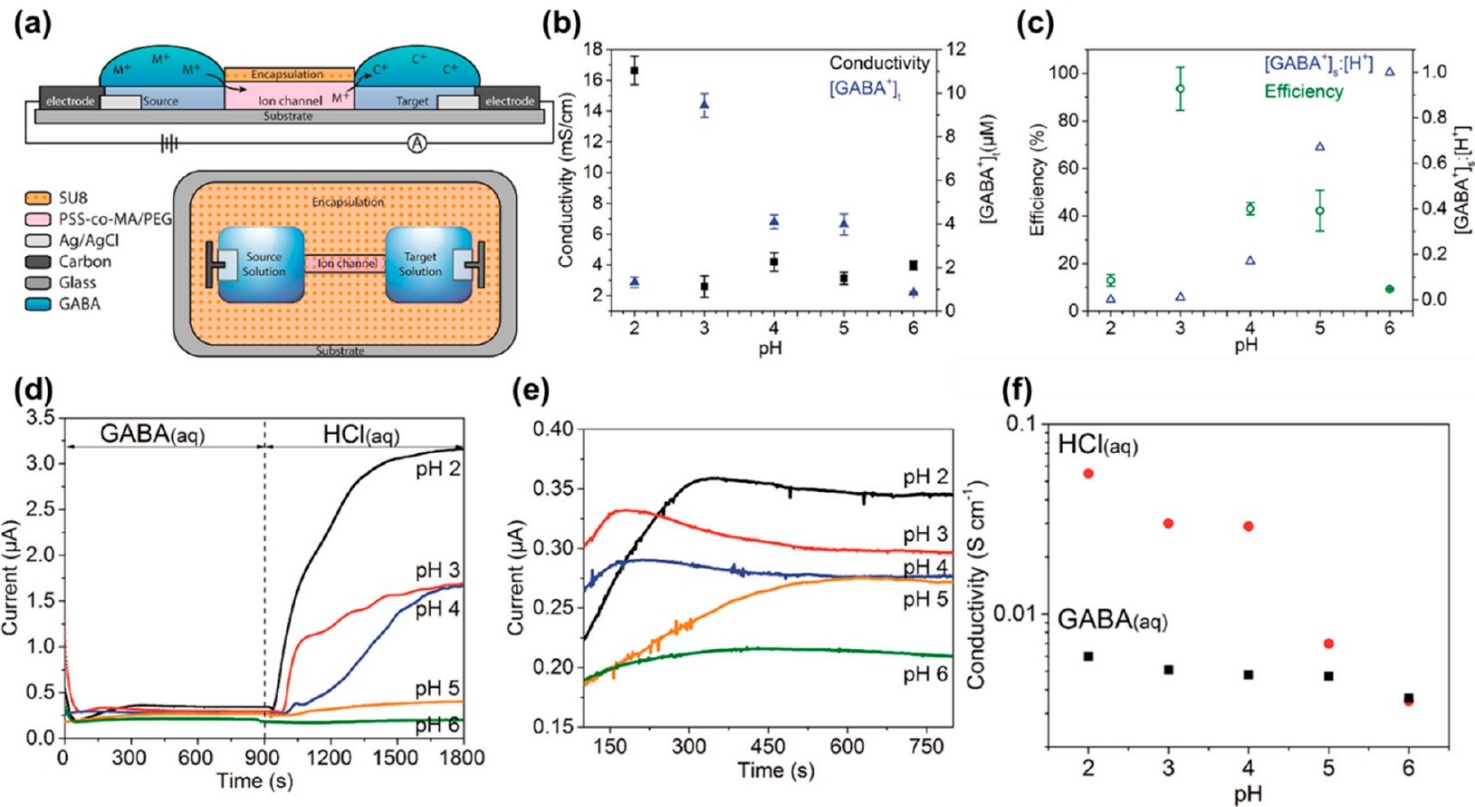
(a) Graphic representation of the organic electronic ion pump (OEIP). Chemical description: (b) support of ionic conductivity for *I*_appl_ = 200 nA and provided [GABA^+^]_t_ at different pH of original GABA solution; (c) support of efficacy of GABA transport and [GABA^+^]_s_/[H^+^] proportion upon pH of original GABA solution. Electrical description: (d) ion pump current vs time toward different pH of reference electrolyte; (e) enlargement of the arc in function for original GABA electrolyte; (f) reliance of ionic conductivity on pH for GABA and HCl reference electrolytes as determined from the steady-state current within the arc by utilizing Ohm’s law and understanding the geometry of the track. Reprinted from ref (97). Copyright 2017 American Chemical Society.

Seitanidou et al.^97^ studied the H dependence of GABA inotropic transportation effects. First, 0.1 M GABA was packed in the original reservoir and pH-adjusted by adding HCl (within pH 2–6), and a steady potential of 10 V was used (Figure 9a). Including both [GABA]_s_ and [GABA]_t_ computed at the individual original pH value, it would resemble the practical integrated ionic conductivity vs initial pH (Figure 9b). The outcomes demonstrate that pH 3 shows the most elevated transport effectiveness for GABA^+^ (Figure 9c). The original reservoir was doped with 0.1 M GABA and pH-modified by adding HCl, and a steady potential of 10 V was used (Figure 9d). Na^+^ is likey to be the prevalent counterion within the poly(4-styrene sulfonic acid-*co*-maleic acid)polyethene glycol (PSS-*co*-MA/PEG) channel from the incorporation phase. Therefore, the height periods marked within Figure 9e show a complicated interaction of Na^+^ for a combination of H^+^ and GABA^+^, with more rapid peak periods typically following toward more heightened [H^+^] into the reference media. It is possible that the conductivity is a part of pH by carrying the sturdy currents at individual pH toward the GABA and HCl media and transforming them to ionic conductivity by employing the PSS network geometry (Figure 9f).

### 3.8 Acetylcholine Importance within the Physiological System

Acetylcholine (ACh) is an essential NT of the human body found in both the CNS and the PNS. In the CNS and the PNS, it can be synthesized by choline acetyltransferase (ChAT) and both ChAT and carnitine acetyltransferase (CarAT), respectively^98^ and stored in the synaptic vesicles and released into the synapse in a calcium-dependent manner. Postsynaptic action binds to receptor proteins such as nicotinic and muscarinic acetylcholine receptors, causing depolarization. ACh is degraded by acetylcholinesterase that alters ACh into inactive metabolites, cholin and acetate. This enzyme rapidly clears free acetylcholine from the synapse, which is essential for appropriate muscle function. Specific neurotoxins are used to prevent acetylcholinesterase. Therefore, foremost, the additional ACh at the neuromuscular junction causes paralysis of the muscle desired for breathing and ending the beating of the heart.^99^

It plays a crucial role in cognitive functions in learning, memory, concentration, and excitement. Its deficiency may lead to brain disorders such as Alzheimer’s disease. It acts as an inhibitory as well as an excitatory neurotransmitter. However, compared to other NTs, such as dopamine and GABA, its role in the nervous system is less understood.

## 4 Overview and History of Sensors

At the beginning of the 20th century, biosensor-based research was started. Two biosensing-based discoveries in 1956 were an oxygen electrode designed by Clark and the glucose sensor in 1962.^100,101^ Professor Leland C. Clark modified electrodes, which was further developed into a glucose sensor that determines oxygen depletion through the performance of glucose oxidase at glucose.^102,103^

The New York Academy of Sciences conference was attended by Professor Leland C. Clark in 1962. With his research experience and publication on oxygen probe development, the professor illustrated that it is possible to construct more effective electrochemical sensors with the addition of enzyme transducers as membrane-enclosed sandwiches.^104,105^ From that time, numerous biosensing devices and technologies have been taking place. Moreover, one can say the sensing era was born.

Sensing-based research principally involves analytical devices which convert responses into proper analytical signals produced within a system.^106^ Each sensor-based device has four essential parts: a sample-like support or analyte, an indicator, a transducer, and a measurement tool.

Several sensors depend on the nature and type of sample and transducer. Sensors that convert chemical information like the concentration of a particular trial within the total configuration of proper analytical signals are called chemical sensors. This type of sensor comprises two primary elements: a chemical detection system, i.e., the sensor, and a physiochemical transducer.^107^ Devices that convert biological signals into processable and sound signals are termed biosensors.^106,108^ In other words, biosensors are those devices that couple biological sensing material into the transducer. In the past few decades, nanosensors have attracted much focus from researchers within the nanoscale region due to the surging need to calculate and detect physiochemical characteristics in hard-reaching industrial and biological systems. Nanosensors can monitor chemical and physical phenomena in hard-reaching areas such as cellular organelles and the measurement of nanoscopic particles industrially and environmentally.^109^ Electrochemical biosensors can be illustrated as self-reliant integrated devices that provide detailed information quantitatively or semiquantitatively.^107^ Electrochemical biosensing strategies determine profitable strength, uncomplicated miniaturization, outstanding recognition boundaries, and minute analyte quantities. Modification of WEs is done to enhance the detection of specific analytes.

The electrochemical detection approach indicates that the electrochemical biosensor is made to discover quick detection, mainly utilized within biocomponents like antibodies and enzymes to alter electrodes. While the actual biocomponents respond with the same marked analyte, the response may be established and estimated; meanwhile, electrical signs are developed, processed by the electronic technique, and then evolve into the data we may keep directly. Standard detection techniques of electrochemical sensors primarily include linear sweep voltammetry (LSV), cyclic voltammetry (CV), and differential pulse voltammetry (DPV). The principles of electrochemical sensors are based on materials such as carbon NM’s recognition of disease biomarkers.^110−112^

## 5 Electrochemical Sensor

**Figure 10:**
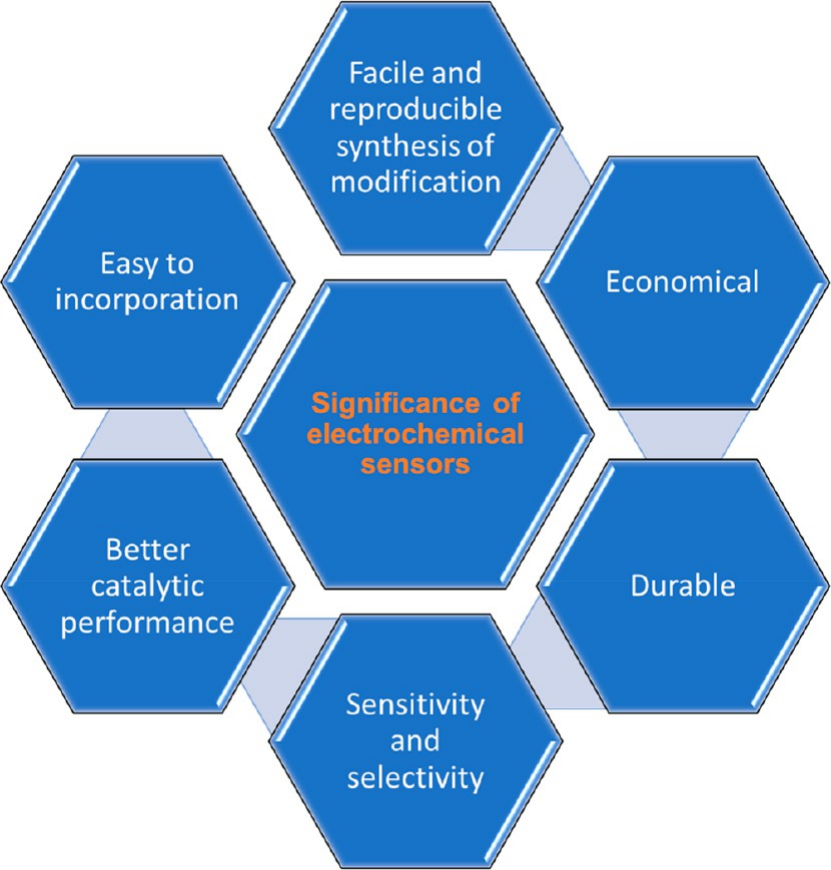
Significance of electrochemical sensors.

In 1906, the invention of electrochemical sensors began with the advancement of the glass electrodes by Cremer.^113^ Haber and Klemensiewicz implemented the essentials of glass electrode potential in 1909 for analytical applications and applied Cremer’s glass electrode basics as the source for diagnostic usages.^102,114^ These sensors provide information regarding the chemical constituents of a system into valuable signals. Two critical parts of sensors are straightforwardly intricate in explaining the contents of the sample. The receptor networks with the analyte into the detection layer, while the transducer converts received info within proper electrical signals.^115^ Electrochemical sensors are broadly used in various industries including traffic, environmental, and biological fields.^116^ These sensors are emerging as reliable analytic techniques that work as an alternative to traditional techniques. Therefore, they act as rising stars in the modern era of detection.^117^ Excellent features of electrochemical sensors are displayed in Figure 10.

### 5.1 Market Worth of Electrochemical Sensors

The glucose sensor is a POC monitoring device that determines and detects glucose levels within the human body and is an electrochemical-based sensor that constitutes a multibillion-dollar market globally. Around 5% of people worldwide have diabetes and utilize this sensor.^118,119^ Consequently, electrochemistry is the dominating criterion for diagnosing various diseases and detecting POC performance, whereas in research and development, optical techniques have established their position.^120,121^ There are two parts of the electrochemical sensor market: diagnosis and monitoring applications. The market value of only POC devices up to 2027 is expected to be worth about $33 billion.^122^

Surging healthcare costs and customers’ demand are probably compelling scientists to produce a novel production of low-priced wearable, integrated, and less-invasive sensors. The sensors must possess the mass output’s flexibility, maintain patients’ well-being, develop pharmaceutical testing, and allow the circulated diagnosis.^120^ Hence, electrochemical-based sensors have immense potential since the system is open to being modified and optimized for detection potential.

### 5.2 Significance of Electrochemical Sensors

Accordingly, electrochemical methods have drawn much concentration in detection since using high-cost tools within the lab surroundings, and expert users’ captive usefulness is revoked.^106,123^ Electrochemical sensors are easily fabricated in various sizes compared to other conventional techniques. Electrochemical techniques are susceptible, allow for rapid analysis due to the less time response, are cost-effective, and are as good as transducer microfabrication technology.^124^ It showed successful extraction of biological information and processing into proper electronic signals even after the direct connection of electronic devices with the biological environment.

The components undergo a biological event that originates electric signals. Lim illustrated that interaction with reactant molecules causes changes in surface structure which helps in detection as it changes their electronic properties.^109^

**Figure 11:**
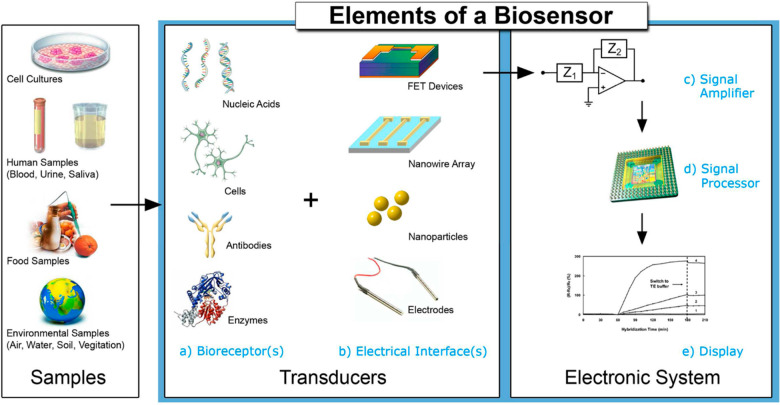
Essentials and designated ingredients of a distinctive biosensor. Reprinted with permission under an open access Creative Common CC BY license from ref (106). Copyright 2008 MDPI.

Figure 11 illustrates the components containing a specific biosensor: (a) receptors, which particularly tie to the analyte; (b) an interface structure, where a typical biological occurrence carries position and provides an upgrade to a sign chosen up by (c) the transducer component; the transducer sign (that would be all from the in-coupling inclination of a laser ray to the current delivered on the electrode) is transformed into an electronic sign and strengthened with a sensor circuit utilizing the practical consideration and transmitted toward processing with, (d) computer software to be transformed to a significant physical parameter representing the operation being studied; ultimately, the resultant abundance has to be introduced via (e) a boundary to the human worker.^106^

Sensors can transduce electrons directly or indirectly. Generally, there is no requirement of any mediator in direct biosensors; they transduce electrons directly through the redox enzyme, an electrocatalyst. When the concentration and quantity of a particular compound are associated with analyte detection, biomolecular sampling is suitable in biochemistry.^125^

### 5.3 Techniques Associated with Electrochemical-Based Sensors

**Figure 12:**
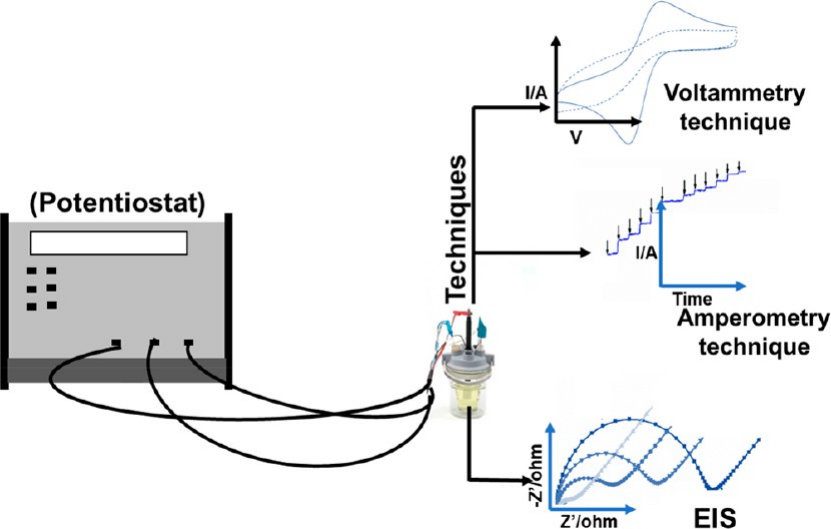
Different electrochemical techniques used in biosensors.

Numerous techniques involved in electrochemical-based sensors are amperometry, potentiometric, and conductometric responses. Mostly potentiometric or amperometry techniques are used to develop electrochemical sensors, while for specific applications, coulometric devices are employed occasionally.^102^ Measurable current, potential, and charge are created in the case of amperometry and potentiometric analysis, while in conductometric analysis, the measurable conductible property is created between two electrodes.^106,126−128^Figure 12 shows the different electrochemical techniques used in biosensors.

#### 5.3.1 Voltammetry Technique

The extent of resulting current (*I*) with varied controlled voltage (*V*) is involved in voltammetric techniques. For electroactive species, there is a direct proportion of bulk concentration of the analyte with the peak value of the record current over a potential linear series.^106,107,126,129^ There are different kinds of voltammetric methods like CV, differential pulse voltammetry (DPV), square wave voltammetry (SWV), and so on, which possess high sensitivity and selectivity. Excitation potential varies with voltammetry time and remains constant in amperometry techniques.^102^

##### 5.3.1.1 Cyclic Voltammetry

In electrochemical detection, CV is the most utilized voltammetric technique. It can study oxidation and reduction potentials of the analyte and reaction mass transfer of the reaction in this type of voltammetry. Peak positions and the shape of the CV curves reveal whether the response is a reversible or irreversible process. To evaluate the electrochemical sensor CV’s probability of selectivity, the first analysis is performed.^130^ Further, the CV is also used for the electrodeposition of the material on the surface of the electrode.

##### 5.3.1.1.1 Slow-Scan Cyclic Voltammetry

Generally, for academic laboratory studies, a slow-scan CV is utilized. This technique, also known as a conventional CV, has a scan rate of fewer than 400 V/s. It is limited to in vitro applications.^131^ The scan rate in the range as low as 0.05 V/s is also used in this CV type. For instance, glutamate was determined by a team of researchers^132^ using NiO nanoparticles. CV was utilized for electrochemical analysis at a scan rate of 0.05 V/s. The sensor exhibits a sensitivity of 11 μA mM^–1^ cm^–2^ and a detection limit of 272 μM.

The time scale for analyzing biological changes is too slow in conventional CV. Therefore, to speed up voltammetry, fast-scan cyclic voltammetry was started by Julian Miller and was popularized by Mark Wightman. To complete a single scan rate in a few milliseconds, they raised the scan rate to quite a few hundred V/s.^133,134^

##### 5.3.1.1.2 Fast-Scan Cyclic Voltammetry

To determine the fast changes in neurotransmitters within the human brain, fast-scan cyclic voltammetry (FSCV) is one of the most popular electrochemical techniques.^135,136^ The scan rates of this technique are 1000 times faster than those of traditional CV, usually 400 V/s with a frequency of 10 Hz. Measurements within subsecond temporal resolution are possible by these scan rates.^137^ Fast-scan voltammetry cannot be performed at conventional larger electrodes like GCE as they take a long time to stabilize a larger background current. Carbon fiber (CF) microelectrodes are suitable electrodes for FSCV as the background current in these electrodes lies in the range of hundreds of nA and has a short time constant.^138^

##### 5.3.1.2 Differential Pulse Voltammetry

DPV is a technique that involves applying a series of regular voltage pulses on a linear ramp potential. A base value of potential is chosen where no faradaic reaction is applied to the electrode, and between pulses, the value of this base potential increases in equal increments. Before using and at the end of the pulse, the current is determined immediately.^139^ It is a sensitive method which allows the simultaneous detection of NTs by utilizing a single pulse.^131^

#### 5.3.2 Amperometry Technique

Measurement of current at constant potential is involved within amperometry. The current is produced during the biochemical reaction due to electrochemical reduction or oxidation of electroactive materials, which is measured continuously in amperometry analysis. The current which is produced is comparable with the concentration of the analyte. Therefore, the analyte concentration can be altered in the vicinity of amperometry sensors. Equilibrium can never be reached in these sensors, although a steady state can be reached. This usually occurs when the constant potential is maintained at the carbon-based WE regarding the reference electrode, which serves as a counter electrode.^107^ This technique measures anti-inference properties.

Clark developed the first amperometry sensor that uses an oxygen electrode,^101^ where silver was oxidized. An equivalent amount of oxygen was reduced into the water, which enters the system through a gas-permeable membrane.^102^

#### 5.3.3 Potentiometric Technique

Determination of potential difference between two reference electrodes separated by a perm-selective membrane in the absence of a significant current or between a reference electrode and an indicator is involved in potentiometric measurements. Potentiometric devices measure the charge potential of the WE compared with the reference electrode (RE) when no significant current is flowing between them.^106,126,129,140^ Potentiometry provides details regarding the ion activity of electrochemical reactions.^106,141^ Finally, potentiometric sensors work near or at equilibrium without any restrictions of electron transportation.

#### 5.3.4 Impedance Technique

In impedance techniques, measurement represents the resistance that is created by the current when a particular voltage is applied. Current is self-possessed against a fixed practical voltage. In a specific frequency range, impedance can be calculated as a ratio of voltage to current and is a vector quantity comprising two independent scalar quantities, namely, resistance and reactance, denoted by *Z*.^142^ In 1998, Bataillard explained this phenomenon concerning an antibody–antigen complex construction that took place upon the exterior of an electrode. The data are plotted as a Nyquist plot where the *x*-axis represents the real part and the *y*-axis represents the imaginary part. This plot is of semicircular form that illustrates the charge transfer process. Impedance can also be described as a Bode plot.

#### 5.3.5 Chronoamperometry

The chronoamperometry technique measures the current when pulse potential is applied to the WE versus the RE.^143^ At the surface of the WE, decreasing or increasing responses occur in diffusion layers of the analyte due to the change in the current.^144^ Single and double potential steps are two general forms in which chronoamperometric experiments are performed. In the former method, the forward potential is applied, and the corresponding current is recorded, whereas in the latter process, the forward potential is used and returned to the starting potential in a given time period.^145^ Selectivity or the anti-interference ability of the analyte is determined by this technique.^146^

Some examples of utilizing electrochemical techniques for determining NTs are briefly discussed below.

**Figure 13:**
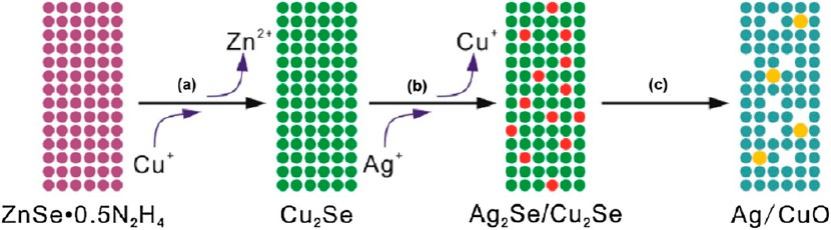
Schematic illustration of AgNP-doped CuO porous nanobelts. (a,b) Complete, partial cation-exchange reaction and (c) thermal oxidation. Reprinted with permission from ref (147). Copyright 2021 Elsevier Ltd.

A AgNP-doped CuO porous nanobelt was synthesized to detect DA by a two-step cation-exchange reaction, followed by in situ thermal conversion, as shown in Figure 13.^147^ CV studies show that AgNPs considerably improve the electrochemical performance of composite toward DA. The performance was analyzed in human serum samples. It showed good reproducibility of up to 3.7% and a linear range of 0.04–10 μM at the lower detection limit of 7 nM. Gu et al.^148^ constructed highly sensitive biofuel cell-based self-powered sensors for the detection of exosomes utilizing glucose dehydrogenase and zeolitic imidazolate modified on an anode and UiO-66-NH_2_ and K_3_[Fe(CN)_6_] on a cathode. The authors obtained the performance of the material by CV. The as-prepared biosensor exhibits a cell voltage of 0.46 V and a maximum power density of 619 μW cm^–2^.

**Figure 14:**
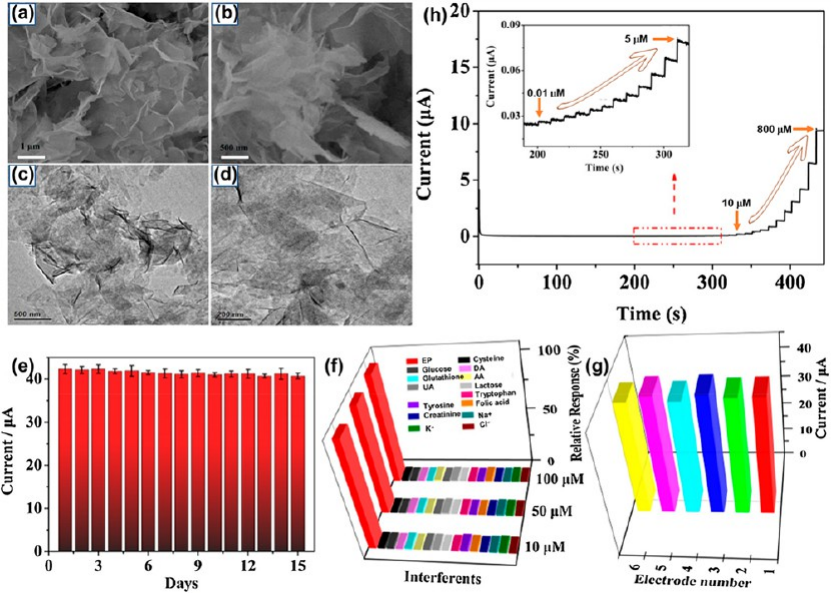
SEM (a,b) and TEM (c,d) images of Ni_6_MnO_8_@C. (e) Stability, (f) selectivity, and (g) observed peak currents of Ni_6_MnO_8_@C consisting of 2 mM epinephrine. (h) Chronoamperometry scans obtained at different concentrations. Reprinted with permission from ref (149). Copyright 2021 Elsevier Ltd.

For the detection of epinephrine, very thin Ni_6_MnO_8_@C was constructed via a hydrothermal and calcinated route having a surface area of 254.26 m^2^ g^–1^. The chronoamperometric technique was utilized to analyze the electrochemical activity of epinephrine on Ni_6_MnO_8_@C. This nanocomposite exhibits high sensitivity in the linear range from 0.01 to 800 μM within the detection limit of 3.33 nM. Figure 14a–d represents SEM and TEM images, and Figure 14e–g illustrates stability, selectivity, and observed peak currents of Ni_6_MnO_8_@C 2 mM EP. Figure 14h showcases the obtained chronoamperometry at different concentrations.

## 6 Different Types of Electrodes for the Analysis of NTs

**Figure 15:**
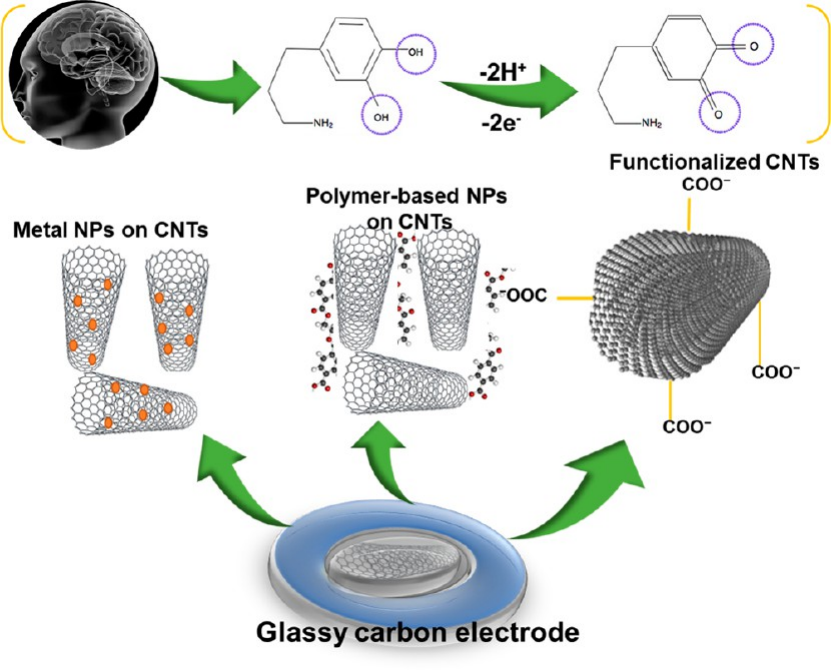
Graphic representation of DA detection using different materials on the GCE.

Carbon-based nanomaterials (CBNMs) have been designed to detect NTs over the past 30 years by operating voltammetry and amperometry. The carbon fiber microelectrode (CFME) is the typical electrode for NT recognition. The CBNM is appropriate for in vivo NT detection because it is biocompatible and comparatively miniature in the exterior area. The source of nanoscale probes has increased need owing to fewer exterior areas that can focus on exact brain areas, which are also minimally intrusive and create somewhat low tissue injury while injected within living organisms. CNTs, CNFs, and carbon nanoplates have all been employed for this objective. Unique electrode substances have also needed novel insulations like glass, epoxy, and polyimide-painted fused silica veins for their structure and use.^150^Figure 15 shows the graphic representation of DA detection using different materials on GCE.

### 6.1 Glassy Carbon Electrodes/Microelectrodes

GCEs are widely utilized inert electrodes in the electroanalysis of NTs due to various characteristics such as easy modification of the surface, electrochemical inertness in the broad potential window, cost-effectiveness, chemical stability, and good conductivity.^151,152^ Specific changes are utilized to facilitate electron transfer processes and increase the electroactive surface area, ultimately improving the GCEs.

For simultaneous detection of DA and serotonin at a concentration of 1 nM, a GC microelectrode was utilized.^153^ The authors obtained a sensitivity of 164 and 110 nA μM^–1^ for DA and serotonin, respectively.

### 6.2 Carbon Fiber Microelectrodes

CFMEs were first introduced by Pujol and colleagues nearly 3 decades ago when they determined the oxidation of NTs using pulse polarography. CFMEs are biocompatible and small in size, usually less than 10 μm. Also, CFMEs do not cause severe damage to tissues during implantation, so they are widely used for in vivo detection of NTs.^154,155^ For detecting NTs, these electrodes are used as standard electrodes in fast-scan voltammetry.

Cao et al.^156^ experimented with activating the surface CFMEs for NT detection. They observed that more oxygen functional groups are introduced in KOH solution, which is advantageous for electron transfer and adsorption. KOH treatment improves the detection limit from 14 ± 4 to 9 ± 2 nM.

### 6.3 CNT-Based Microelectrodes

CNT-based microelectrodes have been studied as options for CFMEs for detecting NTs because they are subtle, show fast electron transfer kinetics, and are better immune to exterior fouling. Wet rotating CNTs with fibers using a thickening polymer produce a thin, consistent fiber incorporated on an electrode. CNT fibers in poly(vinyl alcohol) (PVA) have been utilized as microelectrodes to observe DA, 5-HT, and H_2_O_2_.^150^

The CNT-based electrode showed prompter electron transfer kinetics compared to standard carbon probes because the sp^2^-hybridized CNT network is favorably conductive, and the split ends of CNTs have reactive end plane locations.^157^ CNTs are particularly appealing for creating more miniature electrodes because of the better exterior area-to-volume proportion effects in a sizable electroactive exterior area toward the adsorption of biomolecules. Numerous distinct methods have been designed to incorporate microelectrode shells with CNTs. Dip-covering CNTs upon CFMEs increases sensitiveness, more rapid electron transfer kinetics, and opposition to 5-HT fouling, while the CNTs may aggregate upon the exterior.^158^ Nafion or overoxidized polypyrrole may immobilize CNTs and improve sensitiveness toward DA while applying anionic interferants such as AA.^159^ The considerable subtle CNT-doped CFMEs have aligned CNT woodlands self-decorated upon the exterior, indicating that CNT arrangement is critical. Nevertheless, all of these approaches are challenging to simulate reproducibly, and the electrochemical characteristics of the CF core that may change with distinct wave shapes can influence the electrochemical responses.^160^ Thus, a substantial electrode produced by CNTs would bypass these problems.

Although electrochemical sensors are widely utilized owing to their outstanding properties, certain limitations, such as poor stability and short electrode lifetime, are exhibited in these devices. Different materials are used to modify the surface of electrodes to overcome these issues.

## 7 Role of Incorporation of the Electrode Surface

In the electrochemical system, the role of the electrode surface is crucial. Without discovering different electrodes, several achievements of the electrochemical sensor technology would not be possible. Modification of electrodes enhances the selectivity in various cases where the interaction of reactive species with only desired analytes is needed.^161^ In this regard, we have discussed numerous structures and the role of their surfaces on electrochemical performance in biosensor applications.

### 7.1 Incorporation with Polymers

From the 20th century, polymers have been proven to be essential materials. In 2014, Abed et al. stated that they could develop inert materials, for instance, coatings and containers, into robust active materials by surpassing their potential with valuable mechanical, electrical, and energy storage properties.^162^ Using glass electrodes, polymers influenced the improvement of gas sensors. The CO_2_ sensor where the outward side of a glass electrode was covered with a gas-penetrable polymer was first presented by physiologists Stow and Severinghaus in 2004.^102,163−165^ CPs can act as active materials having remarkable properties that can be transformed as a function of electrochemical potential and are related to their electrochemical nature. CPs were initially synthesized using simple molecules like acetylene and aniline, but now they can be extended to comprise polymers made from heterocycles and aromatic compounds.^166−169^

Conjugated organic polymers can be considered either semiconductors or electrical insulators. They are usually called “electronic polymers” when their electrical conductivity surges considerably from semiconductor systems up to several orders of magnitude.^170^ One such example is aniline-based materials conjugating and semiconducting. Semiconducting materials are fascinating species showing high sensitivity toward numerous analytes countering the conventionally pure conducting species by responding either electrically or optically, and their electronic properties can be changed.^171,172^

CNTs can be considered a semiconducting polymeric structure. Their doping described that the transportation of charge of polymer would be controlled, which results from excitonic electron–hole interactions exhibited in semiconducting materials.^173^ GCE, carbon paste electrodes, and screen-printed electrodes are particularly established as valuable tools to develop both biosensors and electrochemical sensors.^102,174−176^ Further, the role of metallic particles in electrochemical sensors and their properties are comprehensively discussed.

### 7.2 . Incorporation with Metallic Particles

The noble metal NPs (MNPs) having outstanding chemical and physical characteristics are fascinating. They also make the redox reaction kinetically more stable by decreasing the potential of the reaction because of their crucial catalytic properties. MNPs also control the environment because they are highly efficient mass transport catalysts.^177^ They also improve electroactive species’ responses where the oxidation peak’s lowering is provoked by electronic transference between MNPs and redox pair and facilitating the migration of charge via polymer with jumping of charge in conductors.^178,179^

Nanomaterials possess unique physiochemical characteristics like a high ratio of surface area to volume, significant catalytic effects and surface tension force, enhanced mechanical strength and biochemical performance. Currently, an explanation of the high selectivity and sensitivity of nanomaterials in the field of biosensing is given, according to which the reason behind such an extraordinary characteristic is its exclusive property, i.e., quantum property.^180^

1. It catalytically enhances the electrochemical response of DA.
2. It decreases the oxidation peak of NT by electronic change among the redox pair and the MNPs.^61^
3. Most neurochemical recording studies have been carried out with the support of NW or nanotube forests which have a very imposing complete recording measurement of numerous square micrometers.^181^

So far, we have tried to cover the polymer and metallic particles in electrochemical sensors. Further, we have discussed the composite materials’ role and importance.

### 7.3 Incorporation with Composite Material

Composite materials (CMs) are an amalgamation of two or more independent materials consisting of different chemical and physical characteristics that perform synergistically to improve material’s overall performance as a combined system. Enriched CMs can be viewed as more advanced than pure polymers due to significantly improved properties.^182^ The importance of CMs in the mechanical integrity and functionality of materials is described by Choudhary et al.^183^ In material science, these assemblies are very crucial.

A specific class of high**-**performance particles consists of various individual properties that can be considered nanocomposite materials. After doping with inorganic nanomaterials, the characteristics of organic polymers are improved by enhancing the electromagnetic and optical properties of polymers.^184^ These improvements are the consequences of electrocatalytic dispersion of MNPs supported by their structures.^182^ The dispersion of metal nanomaterials is favored by the larger surface area of the polymeric nano- structured matrix. Excellent sensing capabilities are possessed by CPs and MNPs to improve oxidation, charge migration, and electronic transference of electroactive materials.^185^ Here, we have highlighted a significant and facile synthesis route of composite formation, i.e., in situ polymerization and composite formation.

#### 7.3.1 In Situ Polymerization and Composite Formation

Several methods are known for forming composites incorporating MNPs on the polymer matrix. Out of many methods, primary techniques may include (i) reduction of a metal salt with polymer to synthesize nanocomposite, (ii) nanocomposite can be formed with the help of a polymer and a nanomaterial, and (iii) using metal salt and a monomer where metal salt works as oxidizing agent and monomer works as a reducing agent which results in simultaneous formation of metal nanocomposite and polymer. The third is called in situ polymerization and composite formation (IPCF) and is easily controllable.^182^ The driving force of IPCF is oxidative polymerization, where the polymer is formed from monomers and nanomaterial generated from ionic precursors simultaneously.

**Figure 16:**
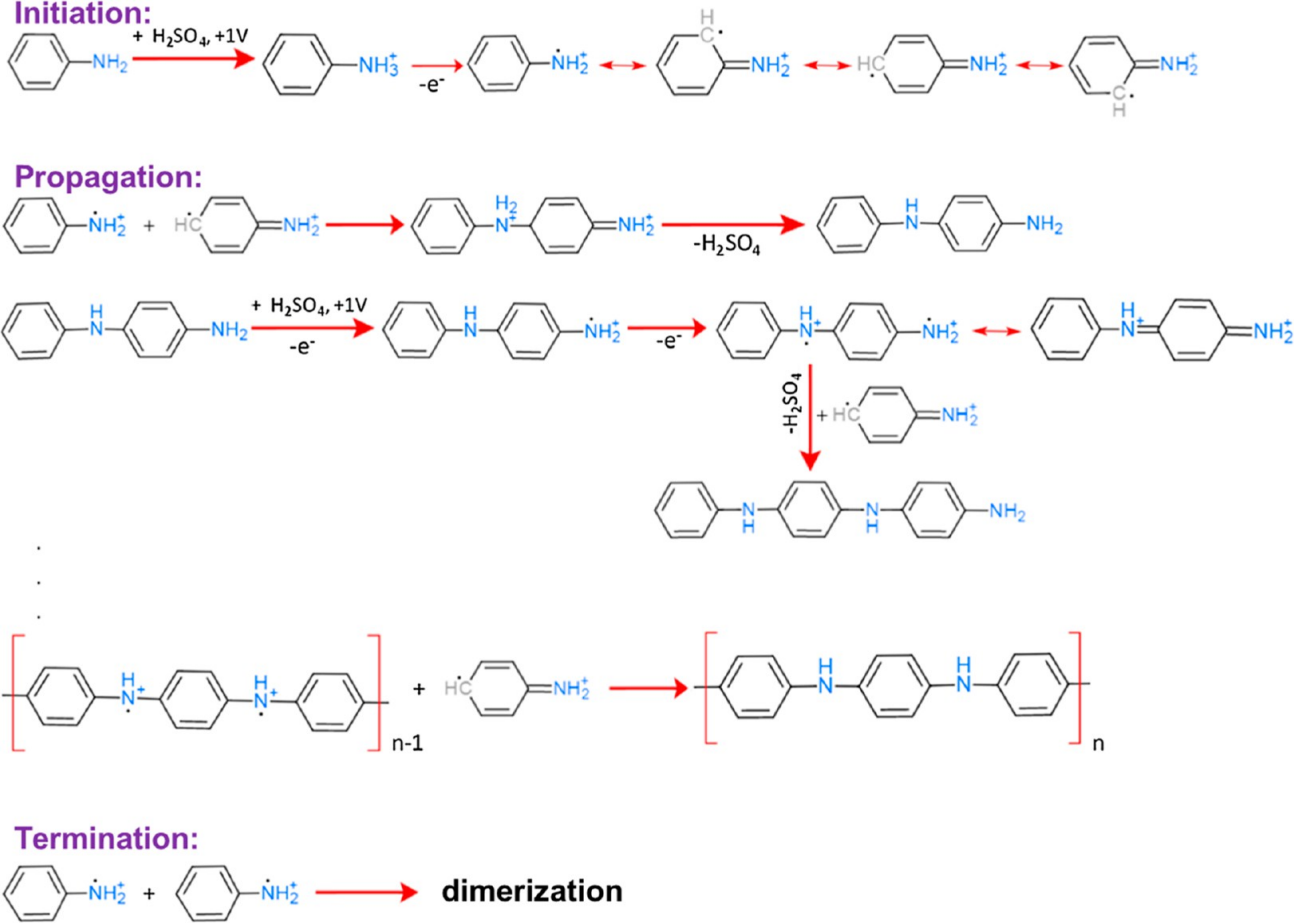
Reaction mechanism for aniline electrochemical polymerization. Reprinted with permission from ref (186). Copyright 2016 Elsevier Ltd.

Furthermore, Figure 16 describes various routes of the oxidative polymerization of aniline. The chain initiation step initiates the process. In this step, the amine group connected to an aniline monomer undergoes oxidation to form a radical cation by releasing a proton and two electrons. The monomer responds with additional monomer at the para-site of the terminal amino group through the electrophilic substitution method. Covalent bonds are formed within monomer molecules that form radical cation that undergoes chain polymerization by the continual growth of chain length after the reaction between radical and monomeric species.^186^

The final step is the chain termination process, where poly condensation occurs due to radical saturation having different chain lengths. The oxidation sites of cation radicals are recombined to form polymers and oligomers of varying lengths. The released electrons produce metal atoms during the polymerization process by reducing metallic ions. The combination of these metal atoms eventually forms NPs, which at the same time are encapsulated by the polymer.^187^

The IPCF process facilitates the interaction between polymer and nanomaterial,^188^ and various aniline-derived polymers such as poly-2aminodiphenylamine (P[2ADPA])^146^ and PANI, can be formed, which acts as supporting conducting polymers. Monomers are crucial in the IPCF process because they release electrons that are utilized to reduce metal particles where reduced metal species coagulate to form MNPs.

An important aspect of analytical methods is robustness in physiological media. Robustness is a parameter which has been assessed in verification investigations of analytical techniques that have been described as the “capability of an analytical approach to deliver fair outcomes in the existence of little modifications within the practical constraints”.^189^ Another notion explains that “robustness could describe the behavior of the analytical process when practical variables intrinsic to the analytical method are little changed”.^190^ A third description suggests that this analytical parameter is “the capability of an analytical method to stay unchanged with little but gradually presented interpretations in process parameters and to indicate its dependability during regular use”. Robustness and ruggedness are often confusing and companion. Nevertheless, there is a difference between these two analytical parameters. The US Pharmacopeia characterized ruggedness as being “the degree of reproducibility of test effects brought with the investigation of the same specimen beneath a type of standard test needs, such as various laboratories, diverse analysts, additional devices, different bunches of reagents, various elapsed assay duration, various assay heats, diverse days, etc.”.^191,192^ This method allowed mixtures from all NT types to elute into small volumes creating intense and symmetric signals and authorizing specific quantifications of small samplings, illustrated with complete blood (100 μL per sampling). An extra robustness-enriching characteristic is automatic filtration/filter back-flushing (AFFL), permitting hundreds of models to be examined without any regions requiring a substitute. For more information regarding the literature, Table 1 lists the synthesis procedure of different nanomaterials and their significance in the field of NTs.

**Table 1:** Synthesis Procedures of Different Nanomaterials and Their Significance in the Field of NTs^a^

type of nanomaterials	synthesis method	detection limit (nM)	sensitivity (μA/μM)	linear range (μM)	detection technique	type of neurotransmitter	ref
Au@PPy/GS	in situ chemical oxidative polymerization	0.01829	16.40	0.0001–5	DPV	DA	(25)
HAu-G	two-step method	50	0.6420	0.08–600	amperometry	DA	(193)
GNP/FTO	e-spray method	0.22 μM	0.004 ± 0.15		CV	DA	(194)
Co_3_O_4_–BiPO_4_	hydrothermal method	1.334 μM		1.71–55	CV	epinephrine	(195)
Ni_6_MnO_8_@C	hydrothermal and calcined procedure	3.33		0.01–800	chronoamperometry	epinephrine	(149)
THH Au–Pd/rGO		0.0012 μM		0.001–1000	DPV	epinephrine	(196)
MnFe_2_O_4_/GCN	sonochemical method	3.1	19.377 μA μM^–1^ cm^–2^	0.1–522.6	CV and DPV	serotonin	(197)
Ni NPs-rGO	atomic layer deposition	0.01 μM		0.02–2	DPV	serotonin	(64)
SnO_2_–SnS_2_/GCE	hydrothermal method	45		0.1–700	CV	serotonin	(198)
MnCr_2_O_4_/MCPE	hydrothermal method	0.034 μM		0.0003–0.0045 mM	CV	norepinephrine	(199)
*p*-amino benzenesulfonic acid/GCE		10	0.455	0.5 to 99.8	CV and DPV	norepinephrine	(200)
CCO nanoplates	soft-template (citrate)-assisted method followed by low-temperature calcinations	30		0.2–3500	CV	ACh	(201)
NiO/MWCNTs/SPE		0.05 μM		0.75–30.0	SWV	norepinephrine	(202)
porous Co_3_O_4_ nanocubes	low-speed chemical synthesis	0.01	20.12 μA mM^–1^ cm^–2^	10–600	CV	l-glutamate	(203)
NiO/GCE	sol–gel method	0.272	11 μA mM^–1^ cm^–2^	0.997–8 mM	CV	l-glutamate	(132)
Pt@erGO/GCE	green synthesis method using a sequential electrochemical method	52		0.25–40	CV	NO	(204)
CuTAPc-MCOF	Schiff base condensation reaction	12.6	29.1 μA mM^–1^ cm^–2^	0.18–17.1	CV	NO	(205)
COF-366-Fe/GA	in situ chemical oxidative polymerization	30	8.8 μA mM^–1^ cm^–2^	0.18–400	CV and amperometry	NO	(206)
nitrogen-ion-implanted WO_3_/ITO	100 keV nitrogen ion implantation process	28	140.57 ± 0.62 μA mM^–1^ cm^–2^	0.1–8000	amperometry	ACh	(207)
Ni–Al LDHs/OMC	simple one-step electrodeposition	42		2–4922	CV	ACh	(208)
nanogold-modified indium tin oxide	touch seed-mediated growth method	0.07 μM	22.9 nA μM^–1^		SWV	ATP	(209)
graphene/Pt		30	0.970	0.03–8.13	DPV	DA	(210)
Ni/NiO@PANI	self-assembled oxidative polymerization	0.087	0.117 nA μM^–1^		CV	epinephrine	(211)

a Au@PPy/GS: Au nanoparticles decorated polypyrrole/reduced graphene oxide; HAu-G: Highly dispersed hollow gold-graphene; GNP/FTO: graphene nanoplatelet-modified fluorine-doped tin oxide electrode; Co_3_O_4_–BiPO_4_: Cobalt oxide-bismuth phosphate; Tetrahexahedral (THH) Au–Pd core–shell nanocrystals on reduced graphene oxide (rGO) nanosheets: THH Au–Pd/rGO; MnFe_2_O_4_/GCN: Manganese ferrite (MnFe_2_O_4_) decorated on graphitic carbon nitride (GCN); Ni NPs-rGO: Ni NPs deposited on rGO; MnCr_2_O_4_/MCPE: MnCr_2_O_4_ nanocomposite modified carbon paste electrode; CCO nanoplates: spinel-type CuCo_2_O_4_ nanoplates; NiO/MWNTs/SPE: nickel oxide based multiwalled carbon nanotube modified screen printed electrode; NiO/GCE: Nickel oxide modified glassy carbon electrode; Pt@erGO/GCE: Pt nanoparticle-decorated electrochemically reduced graphene oxide (erGO)-modified glassy carbon electrode; CuTAPc-MCOF: Metallo-copper phthalocyanine-based covalent-organic framework; COF-366-Fe/GA: Covalent organic frameworks (COF)-366-ferrous/3D graphene aerogel (GA); Ni–Al LDHs/OMC: Ni–Al layered double hydroxides (Ni–Al LDHs) on ordered mesoporous carbon (OMC).

## 8 Other Methods for the Detection of NTs

We briefly discuss other techniques for the diagnosis of NTs, as well.

### 8.1 Optical Method

For the detection of NTs, optical sensors are also utilized.^212−214^ Photoluminescence, surface-enhanced Raman spectroscopy, and optical fiber biosensing are some examples of optical sensing of analytes. In photoluminescence, photons are emitted by material/molecule followed by light stimulation at particular wavelengths. To identify and diagnose the molecules the surface-enhanced Raman spectroscopy utilizes interactions of photons with matter. Several materials such as silver NPs,^213^ hollow-core photonic crystal fiber,^214^ and silver colloids^215^ were designed as appropriate surface-enhanced Raman spectroscopy substrates. The former two materials can cause irreversible aggregations or are toxic. Hence, they are generally not preferred for in vivo diagnosis.^216^ Zhang et al.^212^ fabricated water-soluble silicon NPs to detect DA via the one-pot microwave-assisted method. Fluorescence of synthesized material was uniformly quenched by DA and had no impact on other analytes (proteins, amino acids, peptides Gly, Glu, etc.) in a concentration range of 0.005–10.0 μM and had a lower limit of detection of 0.3 nM. Thus, a susceptible optical sensor was prepared for the diagnosis of DA.

There are a few drawbacks of optical sensors like being intensively sensitive to temperature, interference of background light and pressure, not being suitable for long-term usage due to instability for a long time, and being highly expensive.^217^

### 8.2 Microdialysis

Microdialysis is a considerable standard method for specimen assemblage before cleavage. Microdialysis investigations consist of an inlet pipe and an outlet pipe in a single pole. Perfusate streams via the inlet tube to the probe’s tip that is covered with a semipermeable membrane. Small substances diffuse in and out of the investigation while more significant substances, like proteins, are stopped. Generally, microdialysis is simple and facile to use. However, because of sampling properties, it has limited spatial resolution and long-time delay. The semipermeable probe membrane is attached to the tip in the microdialysis system. Inside this probe, an artificial cerebrospinal fluid is pumped. The NTs are collected in investigations after the diffusion of NTs in the brain under the drive of the concentration gradient.^6^ Equilibrium of molecular distribution is the fundamental nature of the sampling process, which is responsible for the long delay and limited resolution. In addition, the size of the exchange area of the semipermeable membrane also bounds the spatial resolution microdialysis technique. Microdialysis sensors’ performance can be improved to a certain extent by combining microfluidic technology and microdialysis. About 20 min or longer sampling time was taken in 80% of the methods, according to a recent review of microdialysis.^218,219^ Delay in reaching the equilibrium and slow flow rate limits its applicability in neural circuit research.

### 8.3 Optogenetic Control

Optogenetic control of neuronal movement is a unique approach to selectively activating neurons, with overall applications in studying brain processes. Channelrhodopsin-2 (ChR2) is a blue-light started cation channel located within *Chlamydomonas reinhardtii*, which may be inserted in distinct neurons with hereditary manipulations. Upon blue light incitement, ChR2 spreads quickly, and the inward flow of cations shows neuronal excitement.^220,221^ On the other hand, it may control conventional stimulation techniques like electrical or pharmacological stimuli and optical instigation of neurons with millisecond accuracy, allowing targeted activation of a typical kind of neuron within one place. Visual stimulation with ChR2 has been utilized in mammals to comprehend neuronal circuitry that underlies behavior and neurological diseases.^222,223^ Optical stimulus is helpful for tiny sample organisms, like *Drosophila melanogaster*, the fruit fly, because the bipolar electrical compelling electrode is more enormous than the fly’s central nervous system (CNS). *Drosophila* is useful for studying fundamental neurobiological tools because of their simple nervous system, evolutionarily conserved NT pathways, small life cycle, and ease of genetic manipulation.^224^ Utilizing cell-specific booster segments, ChR2 may be inserted within a distinctive kind of neuron in *Drosophila*, and those neurons are triggered through shining of blue light.^225^

Xiao et al.^226^ illustrated the development of pulsed optical stimulus trains within *Drosophila* larval ventral nerve cords upon 5-HT and DA liberation. While *Drosophila* serotonergic and dopaminergic neurons are selectively triggered in vivo, there are significant growths within the part of shoot within the 30–100 Hz content, as well as a nominal growth into the 2–6 Hz spectrum. The experimented pulsed stimuli (10–100 Hz) mimicked the quick-expected firing speeds. They focused upon 5-HT, as 5-HT signaling plays a crucial part within biological procedures like motion and respite, and the 5-HT transporter is a mark for multiple drugs developed to treat psychiatric diseases. The dismissal was estimated utilizing FSCV at a carbon fiber microelectrode embedded into the neuropil of a fly.

### 8.4 Capillary Electrophoresis

Capillary electrophoresis (CE) is a process which splits molecules based upon their electrophoretic mobility, which is mainly defined via their charge and dimensions. CE is inherently suitable as a partitioning strategy for neurochemistry owing to its quick splits, high resolving capability, compliance with tiny sample importance, and direct coupling to a broad assortment of recognition methods.^227^ GABA and other amino acid NTs are usually diagnosed via HPLC and fluorescence or electrochemical recognition, and, as it was noted overhead, these techniques need a large volume of samples. CE does not require such volumes,^228^ instead, low volumes are essential, and the temporal decisiveness is positively enhanced,^229^ mainly when a laser ray causes the fluorescence.^230^

Some benefit of CE is the low volume needed, also other NTs except GABA may be estimated simultaneously, such as Glu, aspartic acid and a few drugs like vigabatrin, and a low limit of detection (LOD) (0.016 μM) has been attained. A distinct drawback concerning GABA investigation via CE is the temperature utilized for derivatization; in a few subjects, 50 °C is required; which signifies an additional therapy method for models, directing to a low temporal resolution. CE-LIFD is now a trustworthy process, completely validated, obtaining unique data on the relations of GABA with different drugs.^231^

### 8.5 Positron Emission Tomography

Positron emission tomography (PET) is a better sensitivity in vivo imaging approach for investigating neural movement. This approach utilizes radioactively labeled substances (tracers) implemented within the bloodstream and metabolized through cells. These tracers decompose and eject positrons that may be noticed and spatially mapped. PET is employed to map cellular operations related to brain movement. For instance, the mixture of PET with voxel-wise investigation permitted mapping of the 5-HT and opioid strategies in the human brain. The PET tracers were a 5-HT transporter tracer and a μ-opioid tracer, [(11)C] carfentanil.^232^ This investigation showed a high degree of overlapping among the indication of 5-HT and opioids within distinct brain areas, for example, the anteromedial thalamus and dorsolateral prefrontal cortex, that are appropriate for regulating discomfort. In another instance, Hooker and co-workers designed a novel approach to observe quick glucose modifications in the human brain, overwhelming the standard low temporal resolve of PET.^233^ In this approach, [^18^F] fluorodeoxyglucose (FDG) was continuously administered intravenously to supply a baseline PET movement and detected quick modifications in glucose metabolism with 5 min of temporal resolution. Many researchers have recently investigated different studies using electrochemical methods, which boosts the knowledge and path in this direction.^234,235^

In overview, PET shows the essential benefit of imagining brain movements concerned with blood flow and molecule metabolism with high acuity. Nevertheless, the disadvantage of this approach is the necessity of producing small radioactive tracers in the bloodstream. Furthermore, it has a low spatial magnification in the millimeter range and temporal resolution at the minute time hierarchy.

## 9 Advantages and Disadvantages of Different Methods

In this regard, Table 2 shows the advantages and disadvantages of different methods used in NT detection.

**Table 2:** Advantages and Disadvantages of Different Methods Used in NT Detection

methods	advantages	disadvantages
electrochemical	real-time detection, simplicity, miniaturization, cost-effective, possibility of continuous analysis on different analytes	to enhance the production of current redox elements are needed, require theoretical stimulation for data analysis
optical	reliable, high sensitivity	surface modification is tough, sensitive to temperature, bulky optical devices are required, need high energy source
PET imaging	vast range of sensitivity from nM to pM and can detect early pathological changes	expertise and resources are required which limits its scalability
	utilizing specific molecular ligands disease mechanism of interest can be interrogated	risks are involved because of repeated radiations
capillary electrophoresis	requires small sample (1–10 nL)	inconsistent retention time
	high separation efficiency	sample may remain stuck to capillary tube
optogenetics	microsecond temporal resolution	costly
	greater potential for multiplexing	toxicity can be involved with exogenous gene

## 10 Conclusion and Future Prospects

Here in this review article, we examined the essential notions connected to a few incorporation techniques and the detailed information about polymer-based MNPs films assuming as primary issues preventing the particle dimensions, their absorbance and diffusion into the polymer matrix, the surface, and eventually, the choice of MNPs mixtures. The prominent emphasis of the study is the brief overview of NTs and their significance in the physiological system. Therefore, the review highlights the history and overview of electrochemical sensors and different techniques to analyze to characterize the proposed materials. Except for the fundamental techniques and broad ideas, the review strives to deliver a complete schematization of the leading technology applications presently in consequence globally. It is worth a thorough analysis of the basic analytes (AA, DA, 5-HT, NO_2_^–^) studied using materials belonging to separate categories to evaluate the efficacy of various hybrid substances. Exciting trends correspond to the prerequisites for sensing these analytes through the diverse incorporated electrodes.

By specifying NE neurons founded upon rhombomeric source and developmental gene terms, we have achieved unparalleled entrance to the primary NE system and demonstrated earlier overlooked assortment into the system. Different determinants of variety, such as discrepancies in inherent genetic programs and extracellular signs, probably contribute to additional heterogeneity within the NE system. The electrodes employed toward the recognition of the different NTs over the years, it is appropriate to indicate that a more significant percentage of the limit of detections were chosen to utilize the DPV owing to its adequate sensitivity corresponded to most voltammetric strategies that topped within lower detection limit in maximum matters. For this cause, it is essential to prioritize the usage of DPV within the hereafter electro-analytical detection of NTs, among other techniques.

Electrochemical detection of analytes, such as glucose nursing, has mainly contributed to enhancing the age of the diabetic patient. As most diabetes is growing globally and healing of the two kinds of diabetes stays unavailable, humankind can aid from advancements in the electrochemical intensive care of glycemia. Eventually, we consider that the current, stunning improvement in the management of the surface of nanostructured composites is possible to maintain curiosity in CP-based materials from an electrocatalytic and electrochemical sensing standpoint, particularly for each electrocatalytic and electrochemical sensing viewpoint graphene-based (ternary) composites. This direction may be already noticed from the current advancement within the numeral of investigations upon this subject.

In the future, safer and more biocompatible NPs should be synthesized for incorporation with sensors. Development in customized sensors can also be achieved by improvement in sensing technology in the case when patients have resistance regarding some specific drugs or biomarkers. Other than this, the commercialization of sensors can be improved with the collaboration of clinicians and electronic expertise with nano scientists. It improves sensing technology and helps improve patients’ everyday life.

## Abbreviations

NT: Neurotransmitter
DA: Dopamine
CNS: Central nervous system
PD: Parkinson’s disease
POC: Point-of-care
WE: Working electrode
GCE: Glassy carbon electrode
ITO: Indium tin oxide
CNTs: Carbon nanotubes
CNFs: Carbon nanofibers
T3: Triiodothyronine
T4: Thyroxine
PNS: Peripheral nervous system
ANS: Autonomous nervous system
SMS: Somatic nervous system
WHO: World Health Organization
CP: Conductive polymers
PANI: Polyaniline
NWAs: Nanowire arrays
GF: Graphene foam
CVD: Chemical vapor deposition
UA: Uric acid
AA: Ascorbic acid
CV: Cyclic voltammetry
EDX: Energy dispersive X-ray spectroscopy
SEM: Scanning electron microscopy
SN: Substantia nigra
PLGA: Poly(lactide-*co*-glycolide)
MCA: Microchamber array
EH: Epinephrine hydrochloride
LSCA/LSCI: Laser speckle contrast analysis/imaging
cAMP: Cyclic adenosine monophosphate
5-HT: 5- hydroxy tryptamine
5-HTP: 5- hydroxytryptophan
SS: Serotonin syndrome
VMAT: Vesicular monoamine transporter
SERT: Serotonin reuptake transporter
5-HIAA: 5-hydroxy indole acetic acid
SSRI: Selective serotonin reuptake inhibitor
SNRI: Serotonin noradrenaline reuptake inhibitor
NE: Norepinephrine
FFN: False fluorescent neurotransmitter
NET: Norepinephrine transporter
VMAT2: Vesicular monoamine transporter
hNET-HEK: Human embryonic kidney cells stably transfected with human NET
Glu: Glutamate
GABA: Gamma-aminobutyric acid
PSS-*co*-MA/PEG: Poly(4-styrenesulfonicacid-*co*-maleicacid) polyethylene glycol
OEIP: Organic electronic ion pump
NO: Nitric oxide
NA: Noradrenaline
NOS: Nitric oxide synthase
NMDA: *N*-methyl-d-aspartate
Ach: Acetylcholine
eNOS: Endothelial NOS
iNOS: Inducible NOS
nNOS: Neuronal NOS
PSD-95: Postsynaptic density
VIP: Vasoactive intestinal polypeptide
ChAT: Choline acetyltransferase
CarAT: Carnitine acetyltransferase
ATP: Adenosine 5- triphosphate
ADP: Adenosine diphosphate
RGCs: Retinal ganglion cells
DPV: Differential pulse voltammetry
SWV: Square wave voltammetry
RE: Reference electrode
CMs: Composite materials
IPCF: in situ polymerization and composite formation
P[2ADPA]: Poly-2aminodiphenylamine
CFME: Carbon fiber microelectrode
PVA: Poly(vinyl alcohol)
Au@PPy/GS: Au nanoparticles decorated polypyrrole/reduced graphene oxide
HAu-G: Highly dispersed hollow gold-graphene
GNP/FTO: Graphene nanoplatelet-modified fluorine-doped tin oxide electrode
Co_3_O_4_–BiPO_4_: Cobalt oxide-bismuth phosphate
THH Au–Pd/rGO: Tetrahexahedral (THH) Au–Pd on reduced graphene oxide
MnFe_2_O_4_/GCN: Manganese ferrite decorated on graphitic carbon nitride
Ni NPs-rGO: Ni nanoparticles deposited on rGO
MnCr_2_O_4_/MCPE: MnCr_2_O_4_ nanocomposite modified carbon paste electrode
CCO nanoplates: Novel spinel-type CuCo_2_O_4_nanoplates
NiO/MWCNTs/SPE: Nickel oxide based multiwalled CNT modified screen printed electrode
Pt@erGO/GCE: Pt nanoparticle-decorated rGO-modified GCE
COF: Covalent-organic framework
CuTAPc-MCOF: Metallo-copper phthalocyanine-based COF
COF-366-Fe/GA: COF-366-ferrous/3D graphene aerogel (GA)
Ni–Al LDHs/OMC: Ni–Al layered double hydroxides on ordered mesoporous carbon
MNPs: Metal nanoparticles
NPs: Nanoparticles
